# Benchmarking miRNA reference genes in B-cell precursor acute lymphoblastic leukemia

**DOI:** 10.1038/s41598-024-77733-8

**Published:** 2024-11-02

**Authors:** Teresa Mack, Tommaso Gianferri, Alexandra Niedermayer, Klaus-Michael Debatin, Lüder H. Meyer, Vera Muench

**Affiliations:** 1 Department of Pediatrics and Adolescent Medicine, Ulm University Medical Center, Ulm, Germany; 2 International Graduate School in Molecular Medicine, Ulm University, Ulm, Germany

**Keywords:** miRNAs, Acute lymphocytic leukaemia, Cancer models

## Abstract

MicroRNAs (miRNAs) play dual roles in acute lymphoblastic leukemia (ALL) as both tumor suppressors and oncogenes, and miRNA expression profiles can be used for patient risk stratification. Precise assessment of miRNA levels is crucial for understanding their role and function in gene regulation. Quantitative real-time polymerase chain reaction (qPCR) is a reliable, rapid, and cost-effective method for analyzing miRNA expression, assuming that appropriate normalization to stable references is performed to ensure valid data. In this study, we evaluated the stability of six commonly used miRNA references (5sRNA, SNORD44, RNU6, RNU1A1, miR-103a-3p, and miR-532-5p) across nine B-cell precursor (BCP) ALL cell lines, 22 patient-derived xenograft (PDX) BCP ALL samples from different organ compartments of leukemia bearing mice, and peripheral blood mononuclear cells (PBMCs) from six healthy donors. We used four different algorithms (Normfinder, ∆CT, geNorm, and BestKeeper) to assess the most stably expressed reference across all samples. Moreover, we validated our data in an additional set of 13 PDX ALL samples and six healthy controls, identifying miR-103a-3p and miR-532-5p as the most stable references for miRNA normalization in BCP ALL studies. Additionally, we demonstrated the critical importance of using a stable reference to accurately interpret miRNA data.

## Introduction

MicroRNAs (miRNAs) are short, approximately 22-nucleotide-long RNAs that post-transcriptionally regulate gene expression, thereby playing significant roles in both physiological and pathological processes^1^. They are transcribed by RNA Polymerase II into long primary miRNAs (pri-miRNAs), which are then processed by the endonuclease DROSHA into ~ 60-nucleotide precursor miRNAs (pre-miRNAs). These pre-miRNAs are transported to the cytoplasm, where the endonuclease DICER produces mature miRNA duplexes. The guide strand of the mature miRNA incorporates into the RNA-induced silencing complex (RISC) with Argonaute proteins, targeting specific messenger RNAs for degradation or translational repression, thereby regulating gene expression post-transcriptionally^1^. As of today, nearly 2,000 human pri-miRNAs are cataloged in the miRBase database (https://www.mirbase.org)^2^, highlighting their broad potential in being involved in physiological and disease situations, particularly cancer and including acute lymphoblastic leukemia (ALL)^3^.

ALL is the most prevalent malignant disease in children and adolescents, with 5-year event-free survival rates exceeding 90% due to the implementation of standardized, intensive chemotherapy regimens^4^. Recently, immunotherapies targeting leukemia-specific antigens have further improved treatment outcomes and reduced chemotherapy-associated side effects^5^. However, relapses still occur and are linked to poor prognosis, underscoring the need for novel treatment strategies^6^.

MiRNAs in ALL can act as oncogenes or tumor suppressors, and their expression profiles are useful for patient risk stratification^7,8^. For example, the miR-497/195 cluster, which is epigenetically regulated in BCP ALL, controls the expression of cell cycle relevant genes and is associated with patient outcomes^9^, and differential miRNA expression might provide information to identify novel therapeutic targets^3^. In *BCR::ABL1*-positive ALL, for instance, downregulated miR-17 ~ 19 is associated with increased levels of the anti-apoptotic protein BCL-2. Targeting BCL-2 has been shown to reduce the proliferation and induce apoptosis of *BCR::ABL*-positive ALL cells^10^.

In the search for novel therapeutic targets and biomarkers, high-throughput technologies have been established, providing omics data with high potential interest. Next-generation sequencing and microarray hybridization are frequently used methods to detect miRNA expression profiles in ALL patients^3^. Both approaches collect reliable and comparable data, assuming the miRNA amount and quality of each sample to be constant. Nevertheless, to re-validate the miRNA expression levels of interest, qPCR is regularly performed, which needs data normalization using a reference gene with a constant expressional level within the cohort of interest^11,12^. The choice of reference gene is crucial to produce reliable data and to allow the comparisons of expression levels across various samples and experiments^13^. In studies analyzing the role of miRNAs in ALL biology and disease progression, a variety of different references have been used^14^. In particular, when profiling ALL-associated miRNAs, the choice of solid references is pivotal to allow data comparisons of studies analyzing the biology of ALL compartmentalization, establishing novel ALL biomarkers, or searching for new therapeutical targets.

To study ALL biology and test new anti-leukemic drugs in preclinical settings, mouse xenograft models, such as the NOD/SCID/huALL model, are frequently used^15^. Transplanting human patient-derived xenograft (PDX) ALL samples into recipient mice resembles the human disease by leukemia manifestation in the spleen, bone marrow (BM), and central nervous system (CNS), thus allowing for the investigation of compartment-specific features of ALL^16,17^. Importantly, in PDX mouse models, cancer histology, immunophenotype, and disease-driver mutations are preserved, enabling the study of leukemia biology across a subset of heterogeneous ALL samples^18–20^.

This study aimed at identifying reliable references in a series of BCP ALL cell lines and primary PDX samples derived from different organ compartments for normalization of miRNA expression data obtained by qPCR. We screened six references frequently used in ALL-associated miRNA studies^9,21–25^ and analyzed the expression in nine ALL cell lines, 22 PDX samples in the identification, and 13 PDX specimens in the validation cohort. We analyzed the stability of used references by four different methods (geNorm^26^, BestKeeper^27^, Normfinder^28^, ∆CT^29^) and identified miR-103a-3p and miR-532-5p to be the most stably expressed miRNA references in ALL samples.

## Results

### Characterization of ALL samples included in the identification cohort

**Fig. 1:**
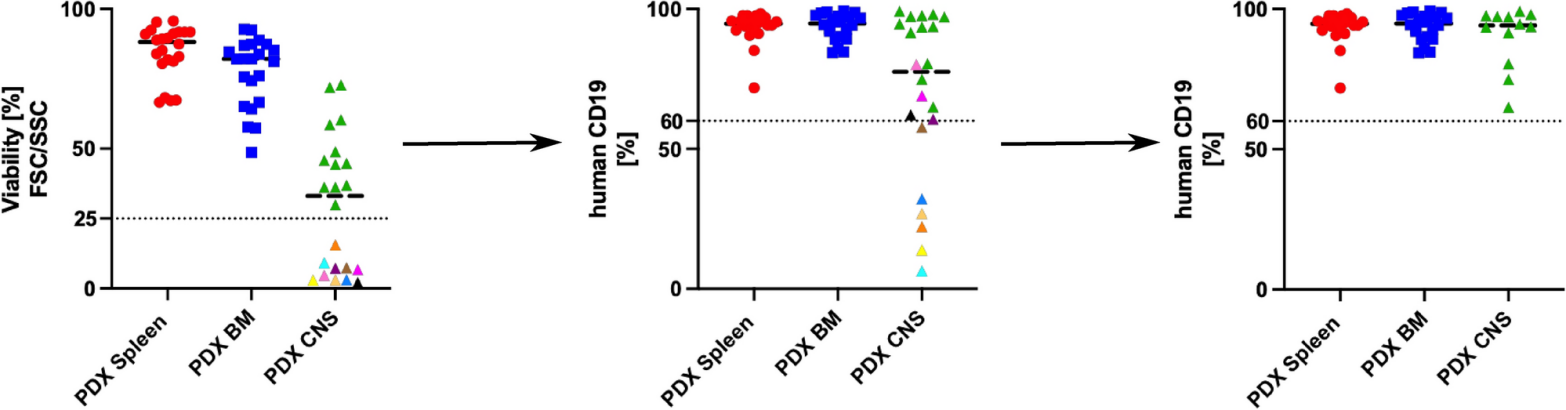
PDX sample selection of the identification cohort. 22 PDX ALL samples were intravenously transplanted into NOD/SCID mice. Upon leukemia-related morbidity, mice were sacrificed, and human leukemia cells were isolated as described previously^8,9^. Samples were stained for human CD19 and murine CD45 and subsequently analyzed according to FSC/SSC criteria determining the percentage of viable cells. Samples with > 25% viable cells (left) were further analyzed according to the percentage of human CD19-positive leukemia cells (middle). 22 BM-, and spleen-, and 12 CNS-derived PDX ALL samples with > 25% viable and > 60% human CD19 were chosen for RNA extraction. PDX samples that were excluded according to FSC/SSC (left) are marked identically when analyzed for CD19-positivity (middle). PDX: patient-derived xenograft; ALL: acute lymphoblastic leukemia; FSC/SSC: forward scatter/side scatter; BM: bone marrow; CNS: central nervous system.

**Fig. 2:**
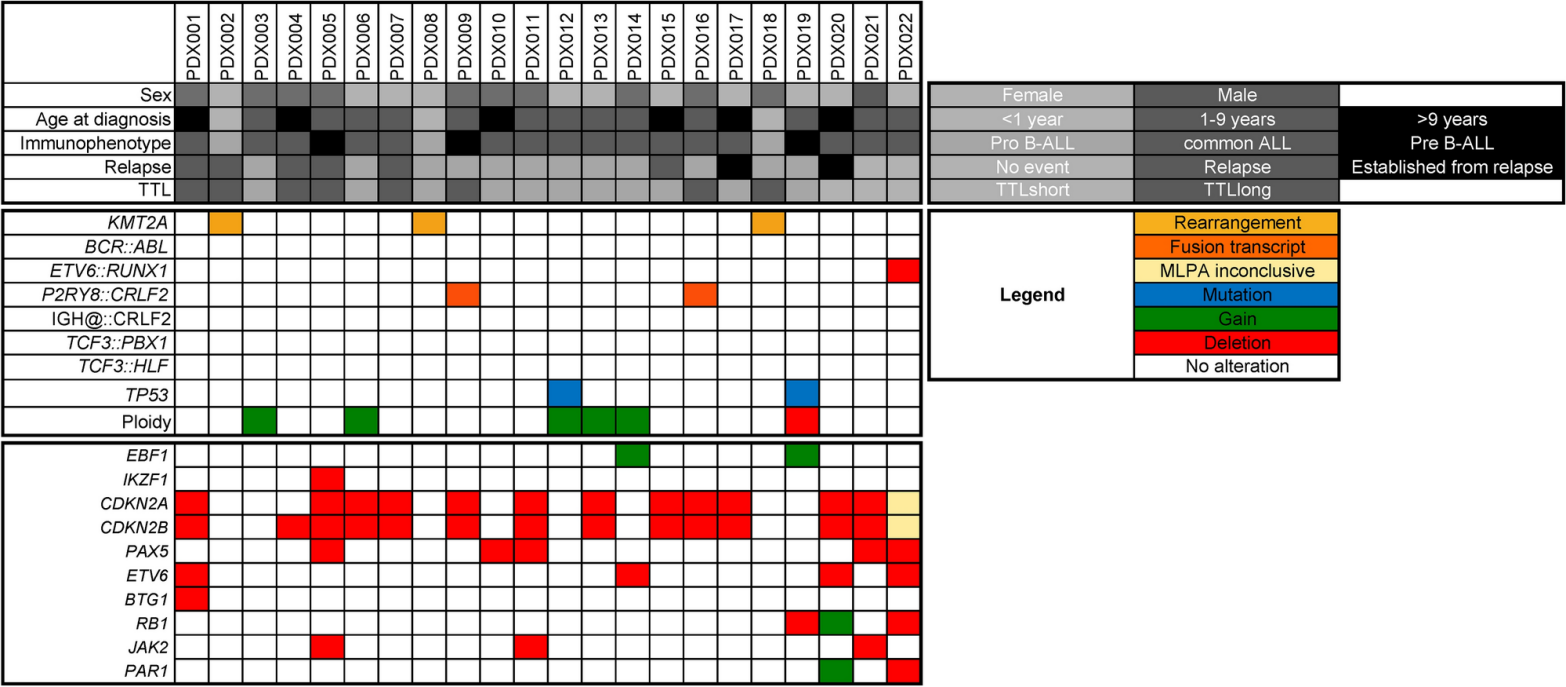
Patient sample characteristics of the identification cohort. Spleen-derived ALL cells were subjected to multiplex ligation-dependent probe amplification, characterizing the samples for frequently detected genetic alterations in ALL. ALL: acute lymphoblastic leukemia; PDX: patient-derived xenograft; TTL: time to leukemia.

MiRNAs have been described to be expressed in a tissue-specific fashion^30^ with varying expression levels between different organ compartments^23^. We aimed at identifying the most stably expressed miRNA references in BCP ALL cell lines (NALM-6, REH, RS4;11, KOPN-8, UoCB6, RCH-ACV, MHH-CALL2, EU3, and HAL-01), and human leukemia PDX samples derived from spleen, BM, and CNS. To investigate the expressional stability of miRNA references in PDX ALL specimens, we intravenously transplanted 22 PDX samples into NOD/SCID mice. Upon the onset of leukemia-related morbidity, leukemia cells were isolated from different organ compartments for further analysis. To validate the engraftment in the spleen, BM, and CNS, we analyzed the samples for the percentage of human CD19 by flow cytometry. For the analysis of miRNA references within these samples, we have chosen samples that were detected > 25% viable according to FSC/SSC criteria and presenting with > 60% human CD19-positive cells (Fig. 1), thereby identifying 22 spleen-, 22 BM-, and 12 CNS-derived leukemia samples which is concordant with previous findings on varying CNS engraftment phenotypes^17^. To detect copy number alterations, insertions, and deletions relevant to ALL biology and patient-risk stratification^31^ in the PDX specimens, we have performed multiplex ligation-dependent probe amplification (MLPA) on spleen-derived ALL cells identifying a heterogenous prevalence of genetic alterations within the identification cohort (Fig. 2).

### Selection of miRNA references

**Fig. 3:**
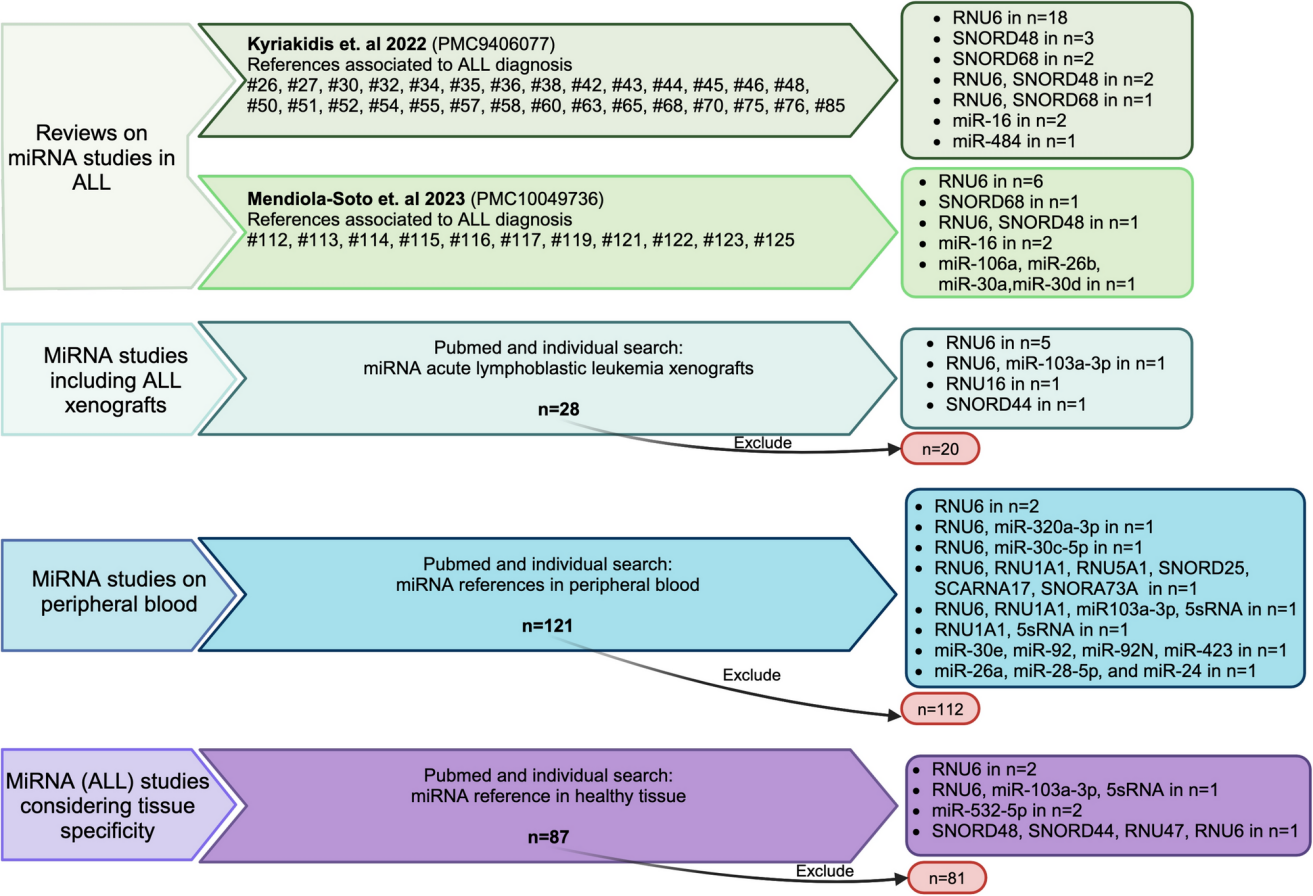
Strategy for selecting miRNA references. Exclusion criteria included review articles, studies on non-human samples or on pathogenic/non-BCP-ALL related diseases, studies focusing on gender-associated miRNAs or tissue not affected by ALL manifestation, studies published in languages other than English, and studies in which no data concerning the use of miRNA references were included. BCP ALL: B-cell precursor acute lymphoblastic leukemia; #number: reference in the corresponding article. Created in BioRender. Meyer, L. (2024) https://BioRender.com/l04p234

For data normalization, miRNA references are accepted that are abundantly expressed, show the same stability and size as miRNAs, and have similar expression levels within the cohort. The group of small nuclear/nucleolar RNAs, ribosomal RNAs, and stably expressed miRNAs meet the criteria of frequently used miRNA references^32–34^. Additionally, based on literature research, we selected relevant miRNA references considering (I) their previous application in ALL studies^14,35^, (II) their role in data normalization in prior ALL xenograft research^9^, (III) their stability in peripheral blood mononuclear cells (PBMCs) from healthy controls^22,36,37^, and (IV) their expression levels across various human tissues allowing taking into account organ-specific ALL manifestation^23,34,37^ (Fig. 3 and Table 1). Applying this strategy, we selected six different miRNA references, including nuclear (RNU6 and RNU1A1), nucleolar (SNORD44), and ribosomal (5sRNA) RNAs, and miRNAs (miR-103a-3p and miR-532-5p) analyzing their expression stability in our cohort samples.

**Table 1:** Strategy for selecting miRNA references.

	RNU6	RNU1A1	SNORD44	5sRNA	miR-103a-3p	miR-532-5p
Reference type	Nuclear	Nuclear	Nucleolar	Ribosomal	miRNA	miRNA
Used in ALL studies	^14,35^		^21^*	^22^*		^23^
Used in ALL xenograft studies	^9,38–42^		^43^		^9^	
Used in miRNA studies on HCs	^44–49^	^36,44,49^		^36,49^		
Analyzed for tissue specificity	^34,50,51^		^37^		^34^	^52^

*Not included in Fig. 3 as not mentioned in reviews; ALL: acute lymphoblastic leukemia, HC: healthy control.

**Fig. 4:**
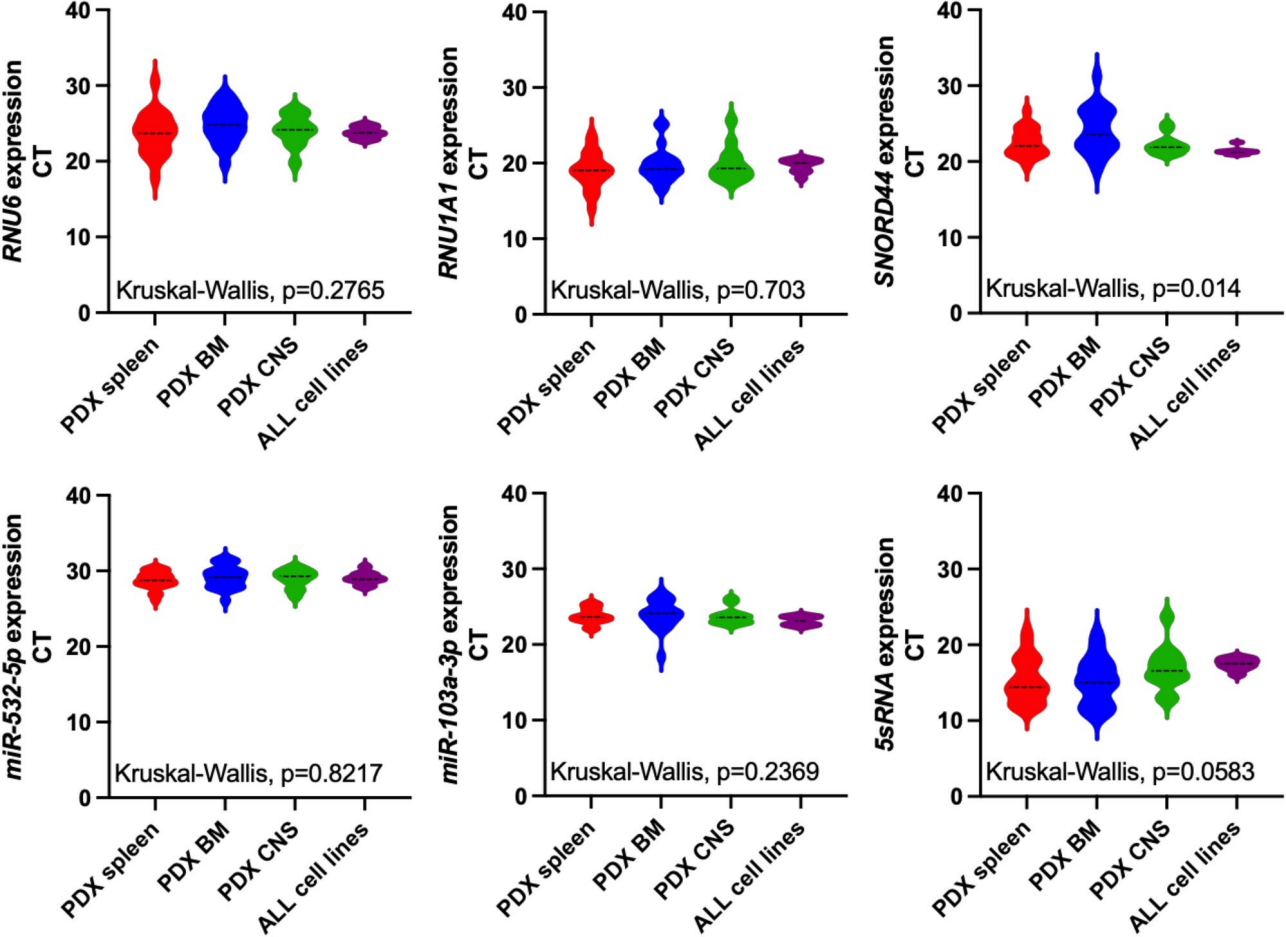
Expression of miRNA references in PDX ALL specimens and ALL cell lines of the identification cohort. The expression of RNU6, RNU1A1, SNORD44, miR-532-5p, miR-103a-3p, and 5sRNA were analyzed in previous-defined PDX ALL samples derived from spleen (n = 22), BM (n = 22), or CNS (n = 12), and in ALL cell lines (n = 9). Kruskal–Wallis test was applied to test whether the median CT values of miRNA references vary amongst the ALL sample groups. PDX: patient-derived xenograft; ALL: acute lymphoblastic leukemia; BM: bone marrow; CNS: central nervous system.

MiRNA references are abundantly expressed in the cohort samples (Fig. 4). Although SNORD44 expression varies significantly across different organ compartments, the expression levels of other references remain stable within the cohort (Fig. 4). The mean CT values ranged from 15.85 for 5sRNA to 28.98 for miR-532-5p (Supplementary Table 1). Next, we analyzed the most stably expressed miRNA reference within the identification cohort (ALL cell lines and PDX ALL specimens) using four different approaches: (1) Normfinder estimating the expression variation amongst cohort samples^28^, (2) ∆CT method of pairs of genes calculating the mean and standard deviations (STD) within the cohort samples and subsequently determining the most stably expressed reference according to the least mean STD^29^, (3) geNorm calculating the average pairwise variations of a set of references^26^, and (4) BestKeeper calculating the STD of the geometric means of the samples per reference finally identifying the most stably expressed reference according to the least deviation^27^. Finally, we applied RefFinder^53^ to rank the reference genes according to their performance in the different algorithms.

**Fig. 5:**
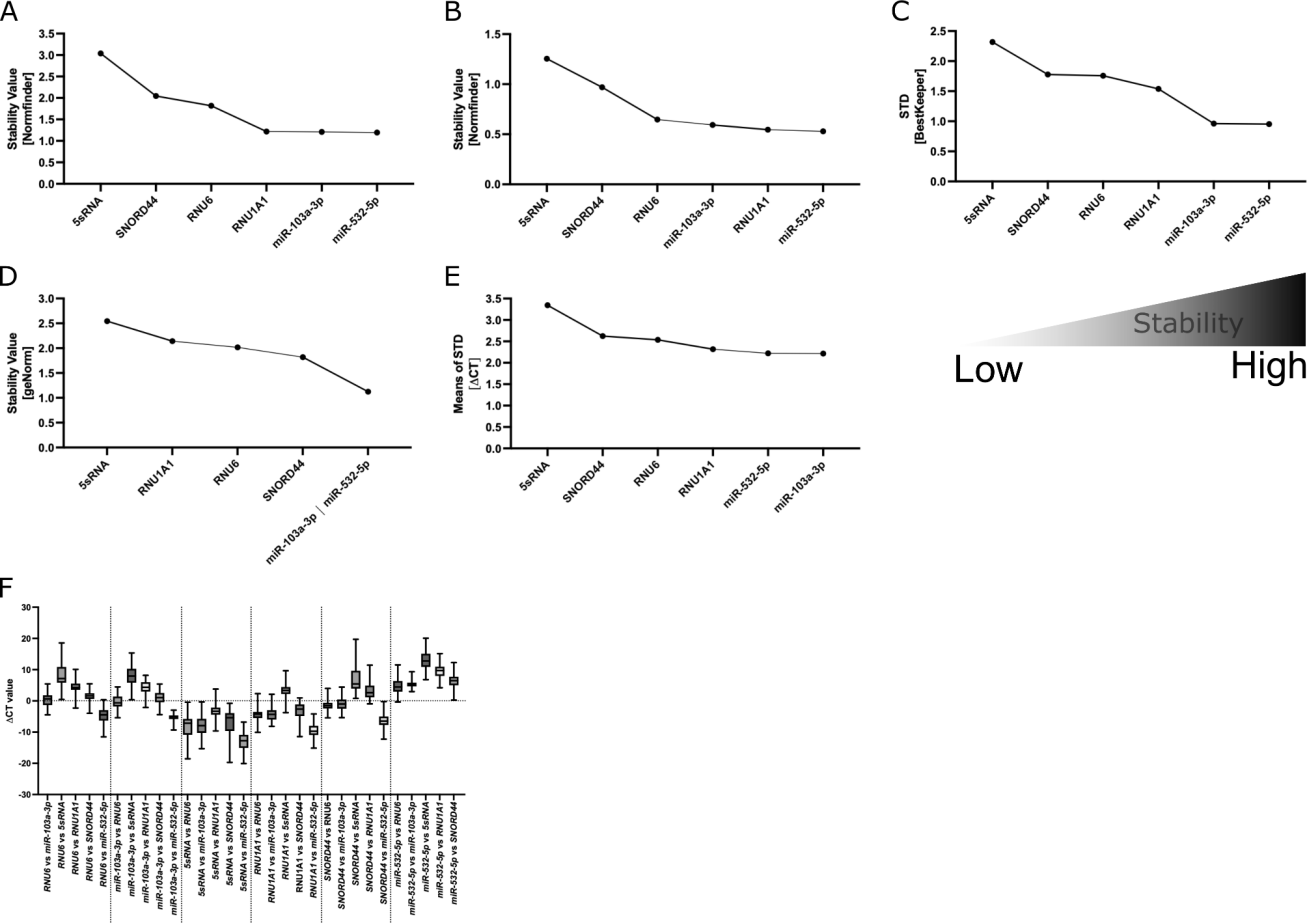
Stability of miRNA references in PDX ALL samples and ALL cell lines of the identification cohort. Expression stability within the cohort samples was analyzed applying the Normfinder (**A** and **B**; **A**: samples clustered in 1 group; **B**: samples sorted in 4 groups according to spleen-, BM-, CNS-derived PDX samples, and ALL cell lines), BestKeeper (**C**), geNorm (**D**), or ∆CT algorithm (**E** and **F**). For **A**-**E**: decreasing values indicate increasing stability. **F**: ∆CT calculations based on CT[reference 1] – CT[reference 2]; mean CT values with min to max whiskers are shown. PDX: patient-derived xenograft; ALL: acute lymphoblastic leukemia.

When Normfinder was used, miR-532-5p followed by miR-103a-3p were identified to be the most stably expressed miRNA references (Fig. 5A). Interestingly, when we applied grouping to our samples (4 groups, namely cell lines, PDX spleen, PDX BM, and PDX CNS), the Normfinder algorithm again identified miR-532-5p as having the best stability value across the groups (Fig. 5B). In the next step, we applied the BestKeeper algorithm. Consistent with the Normfinder data, miR-532-5p, followed by miR-103a-3p, showed the least STD (Fig. 5C). Further, we applied the geNorm algorithm, identifying that the use of miR-532-5p along with miR-103a-3p was best suitable for qPCR data normalization (Fig. 5D). Last, we applied the ∆CT identifying miR-103a-3p (mean STD: 2.215) followed by miR-532-5p (mean STD: 2.220) as the most stable miRNA references within the ALL sample cohort (Fig. 5E-F and Table 2).

**Table 2:** Mean of ∆CT values and STD of PDX ALL samples and ALL cell lines of the identification cohort.

	Mean	STD	Mean STD
RNU6 vs miR-103a-3p	0.3925	2.339	
RNU6 vs 5sRNA	8.302	3.8	
RNU6 vs RNU1A1	4.632	2.233	
RNU6 vs SNORD44	1.361	1.89	
RNU6 vs miR-532-5p	− 4.826	2.416	2.536
miR-103a-3p vs RNU6	− 0.3925	2.339	
miR-103a-3p vs 5sRNA	7.909	3.297	
miR-103a-3p vs RNU1A1	4.24	2.252	
miR-103a-3p vs SNORD44	0.9681	2.066	
miR-103a-3p vs miR-532-5p	− 5.219	1.123	2.215
5sRNA vs RNU6	− 8.302	3.8	
5sRNA vs miR-103a-3p	− 7.909	3.297	
5sRNA vs RNU1A1	− 3.67	2.268	
5sRNA vs SNORD44	− 6.941	4.189	
5sRNA vs miR-532-5p	− 13.13	3.166	3.344
RNU1A1 vs RNU6	− 4.632	2.233	
RNU1A1 vs miR-103a-3p	− 4.24	2.252	
RNU1A1 vs 5sRNA	3.67	2.268	
RNU1A1 vs SNORD44	− 3.271	2.702	
RNU1A1 vs miR-532-5p	− 9.458	2.126	2.316
SNORD44 vs RNU6	− 1.361	1.89	
SNORD44 vs miR-103a-3p	− 0.9681	2.066	
SNORD44 vs 5sRNA	6.941	4.189	
SNORD44 vs RNU1A1	3.271	2.702	
SNORD44 vs miR-532-5p	− 6.187	2.268	2.623
miR-532-5p vs RNU6	4.826	2.416	
miR-532-5p vs miR-103a-3p	5.219	1.123	
miR-532-5p vs 5sRNA	13.13	3.166	
miR-532-5p vs RNU1A1	9.458	2.126	
miR-532-5p vs SNORD44	6.187	2.268	2.220

PDX: patient-derived xenograft; ALL: acute lymphoblastic leukemia; STD: standard deviation.

**Fig. 6:**
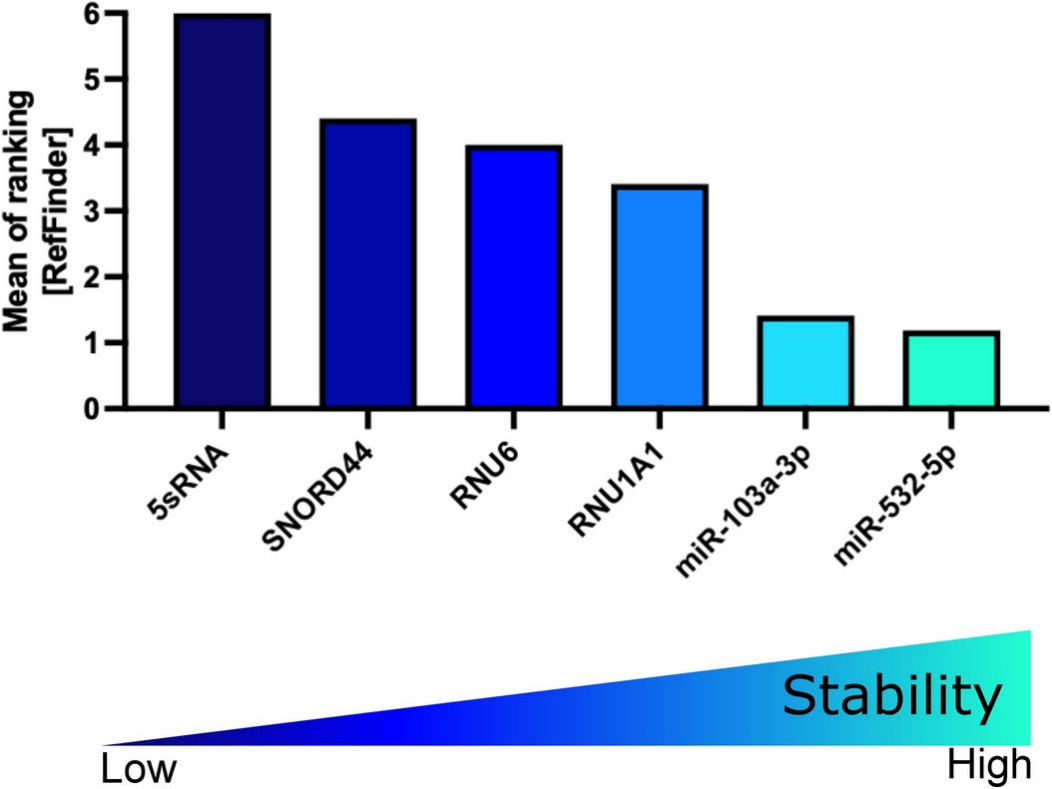
RefFinder on PDX ALL samples and ALL cell lines of the identification cohort. Mean of ranking according to the Normfinder, BestKeeper, geNorm, and ∆CT method applying the RefFinder algorithm. Decreasing values indicate increasing stability. PDX: patient-derived xenograft; ALL: acute lymphoblastic leukemia.

To sum up, the RefFinder algorithm was applied to finally rank the performance of miRNA references. The geometric mean of ranking values identified miR-532-5p followed by miR-103a-3p as the most stable miRNA references that can be used for data normalization of qPCR studies in the identification cohort of ALL samples (Fig. 6 and Table 3).

**Table 3:** RefFinder ranking of PDX ALL samples and ALL cell lines of the identification cohort.

miRNA reference	Rank				
	RefFinder	Normfinder [Stability Value]	BestKeeper [STD]	geNorm [Stability Value]	∆CT [Means of STD]
miR-532-5p	1	1.194	0.954	1.124^★^	2.22
miR-103a-3p	2	1.209	0.963	1.124^★^	2.216
RNU1A1	3	1.218	1.538	2.142	2.317
RNU6	4	1.818	1.756	2.017	2.536
SNORD44	5	2.046	1.777	1.819	2.623
5sRNA	6	3.038	2.318	2.543	3.344

*Best performance in data normalization using miR-532-5p and miR-103a-3p in combination according to geNorm algorithm. PDX: patient-derived xenograft; ALL: acute lymphoblastic leukemia; STD: standard deviation.

### Influence of sample type on reference gene

**Fig. 7:**
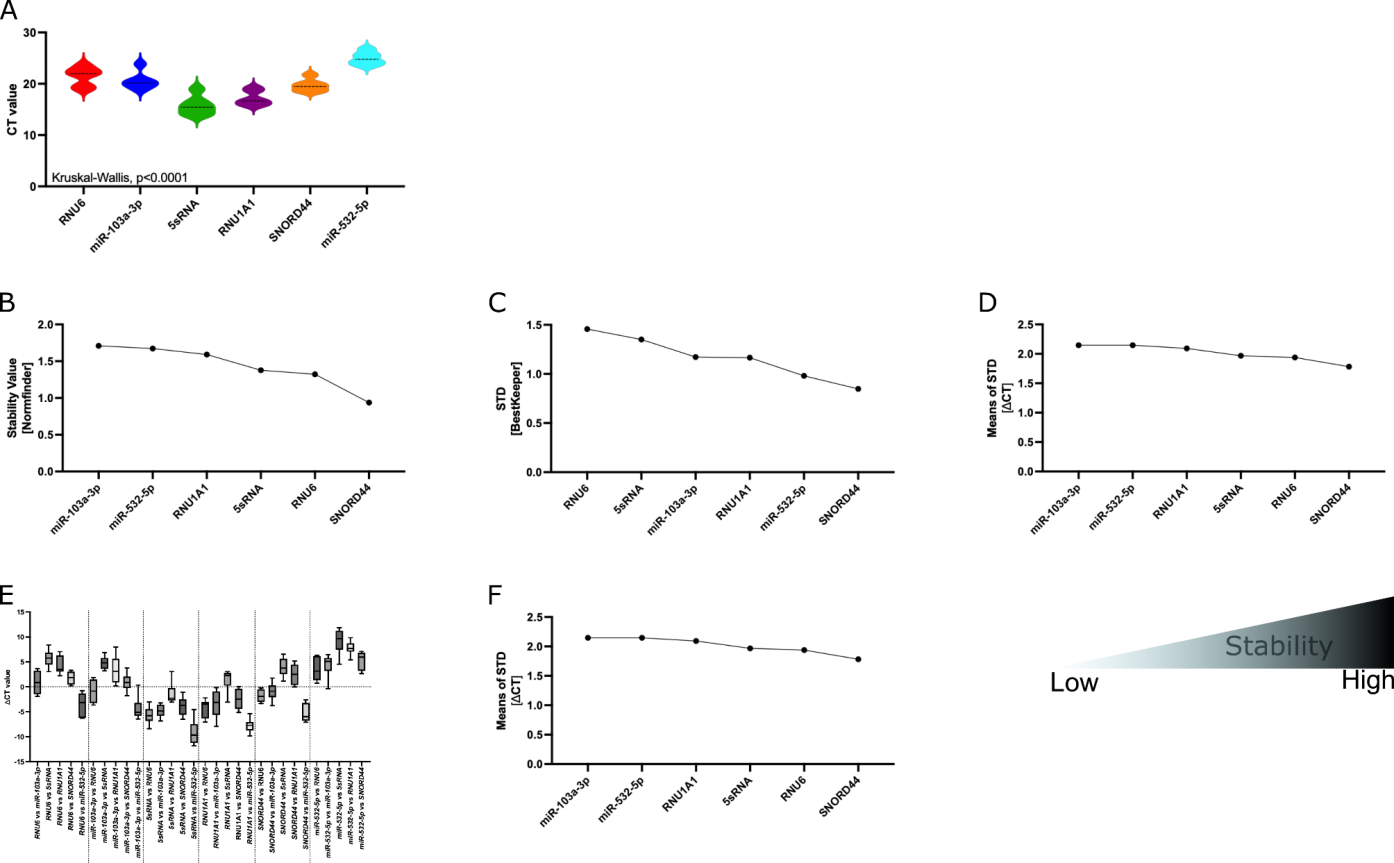
Expression and stability of miRNA references in healthy controls of the identification cohort. (**A**) Expression of miRNA references are depicted as CT values. Expression stability within healthy controls was analyzed by applying the Normfinder (**B**), BestKeeper (**C**), ∆CT (**D** and **E**), or geNorm algorithm (**F**). For **A**-**D** and **F**, decreasing values indicate increasing stability. **E**: ∆CT calculations based on CT[reference 1] – CT[reference 2]; mean CT values with min to max whiskers are shown.

**Fig. 8:**
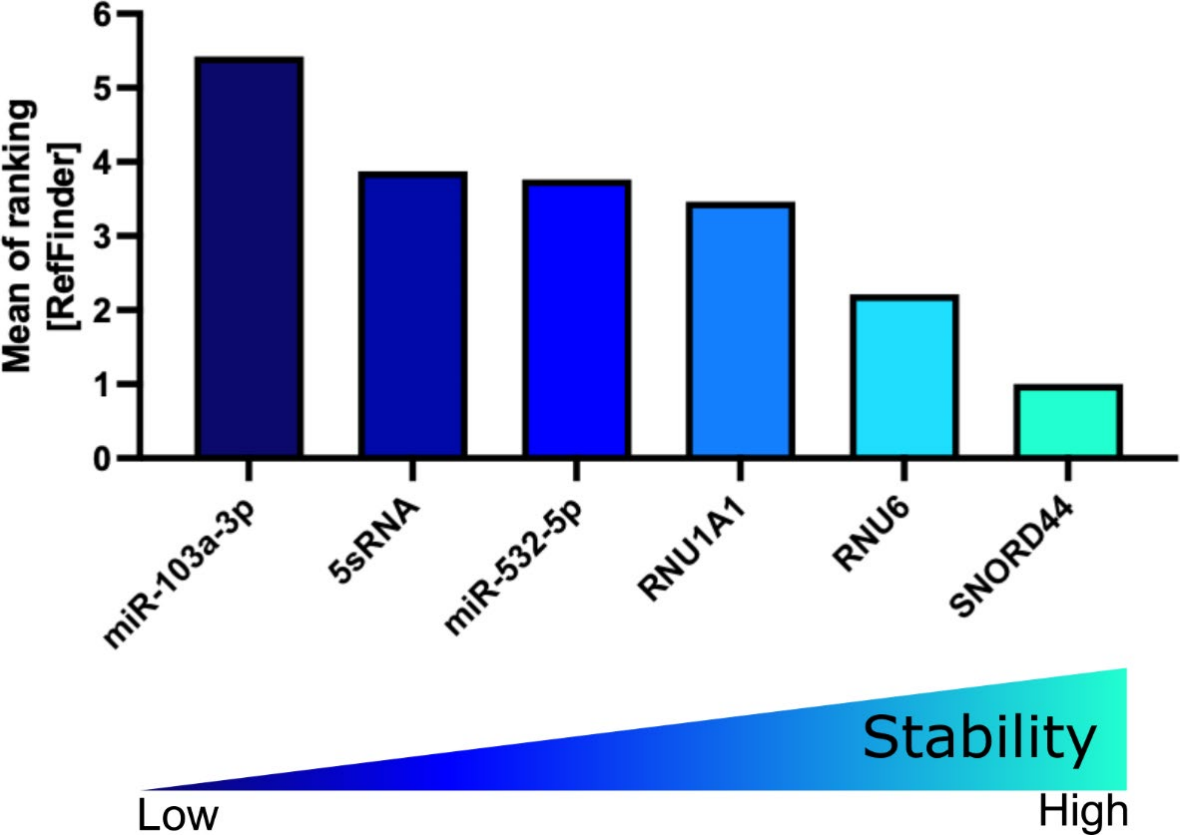
RefFinder on healthy controls of the identification cohort. Mean of ranking according to the Normfinder, BestKeeper, geNorm, and ∆CT method applying the RefFinder algorithm. Decreasing values indicate increasing stability.

Next, we evaluated the expression levels of miRNA references in peripheral blood mononuclear cells (PBMCs) of 6 healthy controls (HCs; 3 female and 3 male). The mean CT values ranged from 15.75 for 5sRNA to 24.95 for miR-532-5p (Supplementary Table 2). We identified the miRNA references heterogeneously expressed in PBMCs of HCs, with SNORD44 showing the smallest STD of 1.185 (Fig. 7A). Accordingly, when applying either the Normfinder (Fig. 7B), BestKeeper (Fig. 7C), or ∆CT algorithm (Fig. 7D, E, and Table 4), SNORD44 was identified as the most stable reference to be used in miRNA studies on PBMCs from HCs. The expression of SNORD44 and RNU6 were detected as reliable references in PBMCs when the geNorm algorithm was used (Fig. 7F). These data were further substantiated when the RefFinder algorithm was applied, identifying SNORD44 as the most stable reference to be used in miRNA studies on PBMCs derived from HCs (Fig. 8 and Table 5).

**Table 4:** Mean of ∆CT values and STD of PBMCs from healthy controls of the identification cohort.

	Mean	STD	Mean STD
RNU6 vs miR-103a-3p	0.8783	2.418	
RNU6 vs 5sRNA	5.7	1.761	
RNU6 vs RNU1A1	4.268	1.867	
RNU6 vs SNORD44	1.797	1.244	
RNU6 vs miR-532-5p	− 3.505	2.406	1.9392
miR-103a-3p vs RNU6	− 0.8783	2.418	
miR-103a-3p vs 5sRNA	4.822	1.295	
miR-103a-3p vs RNU1A1	3.39	2.789	
miR-103a-3p vs SNORD44	0.9183	1.809	
miR-103a-3p vs miR-532-5p	− 4.383	2.424	2.147
5sRNA vs RNU6	− 5.7	1.761	
5sRNA vs miR-103a-3p	− 4.822	1.295	
5sRNA vs RNU1A1	− 1.432	2.28	
5sRNA vs SNORD44	− 3.903	1.907	
5sRNA vs miR-532-5p	− 9.205	2.596	1.9678
RNU1A1 vs RNU6	− 4.268	1.867	
RNU1A1 vs miR-103a-3p	− 3.39	2.789	
RNU1A1 vs 5sRNA	1.432	2.28	
RNU1A1 vs SNORD44	− 2.472	2.085	
RNU1A1 vs miR-532-5p	− 7.773	1.441	2.0924
SNORD44 vs RNU6	− 1.797	1.244	
SNORD44 vs miR-103a-3p	− 0.9183	1.809	
SNORD44 vs 5sRNA	3.903	1.907	
SNORD44 vs RNU1A1	2.472	2.085	
SNORD44 vs miR-532-5p	− 5.302	1.865	1.782
miR-532-5p vs RNU6	3.505	2.406	
miR-532-5p vs miR-103a-3p	4.383	2.424	
miR-532-5p vs 5sRNA	9.205	2.596	
miR-532-5p vs RNU1A1	7.773	1.441	
miR-532-5p vs SNORD44	5.302	1.865	2.1464

PBMC: peripheral blood mononuclear cells; HC: healthy controls; STD: standard deviation.

**Table 5:** RefFinder ranking of HCs of the identification cohort.

miRNA reference	Rank				
	RefFinder	Normfinder [Stability Value]	BestKeeper [STD]	geNorm [Stability Value]	∆CT [Means of STD]
SNORD44	1	0.937	0.848	1.243^★^	1.782
RNU6	2	1.322	1.457	1.243^★^	1.939
RNU1A1	3	1.59	1.166	1.732	2.094
miR-532-5p	4	1.673	0.98	1.818	2.146
5sRNA	5	1.377	1.35	1.945	1.968
miR-103a-3p	6	1.709	1.172	2.012	2.147

*Best performance in data normalization using SNORD44 and RNU6 in combination according to geNorm algorithm. HCs: healthy controls; STD: standard deviation.

**Fig. 9:**
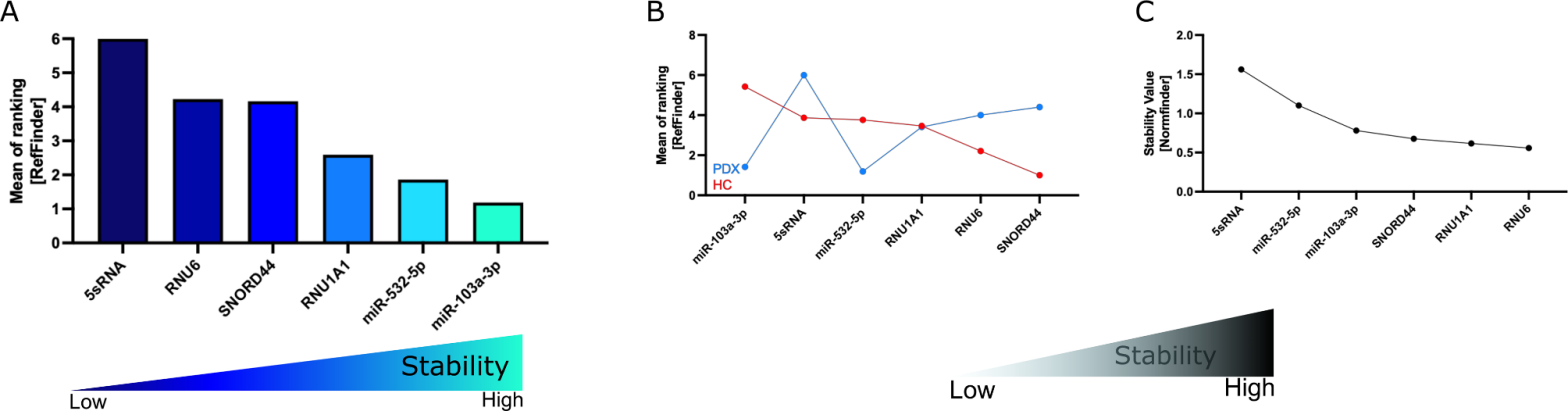
Influence of sample type on miRNA reference stability in the identification cohort. (**A**) Mean of ranking according to the Normfinder, BestKeeper, geNorm, and ∆CT method applying the RefFinder algorithm to the combined cohort of HCs and PDX ALL samples (BCP ALL cell lines, PDX ALL samples including spleen, BM, and CNS). (**B**) Combined graph of RefFinder results of PDX ALL samples (including cell lines and PDX specimens) and HCs as depicted in Fig. 6 and Fig. 8, respectively. (**C**) Stability values according to the Normfinder algorithm applying grouping (HCs and PDX) to the combined cohort. Decreasing values indicate increasing stability. BCP ALL: B-cell precursor acute lymphoblastic leukemia; PDX: patient-derived xenograft; HC: healthy control.

Interestingly, when we included the data of PBMCs into the ALL sample cohort (cell lines and PDX) and applied the RefFinder algorithm, we identified miR-103a-3p followed by miR-532-5p as the most stable references (Fig. 9A and Table 6). Importantly, according to the RefFinder ranking of PDX ALL samples and HCs (both groups considered separately; Fig. 9B), the choice of reference gene has a more pronounced effect on the ALL samples, as evidenced by larger score variations for some references, including miR-103a-3p and miR-532-5p. Moreover, to analyze inherent differences between samples derived from ALL specimens and HC, we used the Normfinder algorithm applying grouping to our samples (ALL and HC) and identified stable RNU6 and RNU1A1 levels (Fig. 9C). This demonstrates low expressional variation within sample types when using RNU6 or RNU1A1 for data normalization, also reflected by low RefFinder score variations of these miRNA comparing ALL and HC specimens (Fig. 9B).

**Table 6:** RefFinder ranking of ALL cell lines, PDX ALL samples, and HCs of the identification cohort.

miRNA reference	Rank				
	RefFinder	Normfinder [Stability Value]	BestKeeper [STD]	geNorm [Stability Value]	∆CT [Means of STD]
miR-103a-3p	1	1.246	1.145	1.277^★^	2.244
miR-532-5p	2	1.309	1.227	1.277^★^	2.272
RNU1A1	3	1.229	1.573	2.141	2.32
SNORD44	4	1.972	1.816	1.85	2.579
RNU6	5	1.765	1.821	2.025	2.507
5sRNA	6	3.044	2.239	2.546	3.356

*Best performance in data normalization using miR-103a-3p and miR-532-5p in combination according to geNorm algorithm. HCs: healthy controls; STD: standard deviation.

### Validation cohort

To validate miR-532-5p and miR-103a-3p as reliable references in BCP ALL studies, we additionally analyzed 13 PDX ALL samples with viability exceeding 25% based on FSC/SSC and over 60% positivity for human CD19. Due to poor RNA quality, three samples were excluded, leaving 12 spleen-, 12 BM-, and 7 CNS-derived ALL samples for validation (Supplementary Fig. 1).

**Fig. 10:**
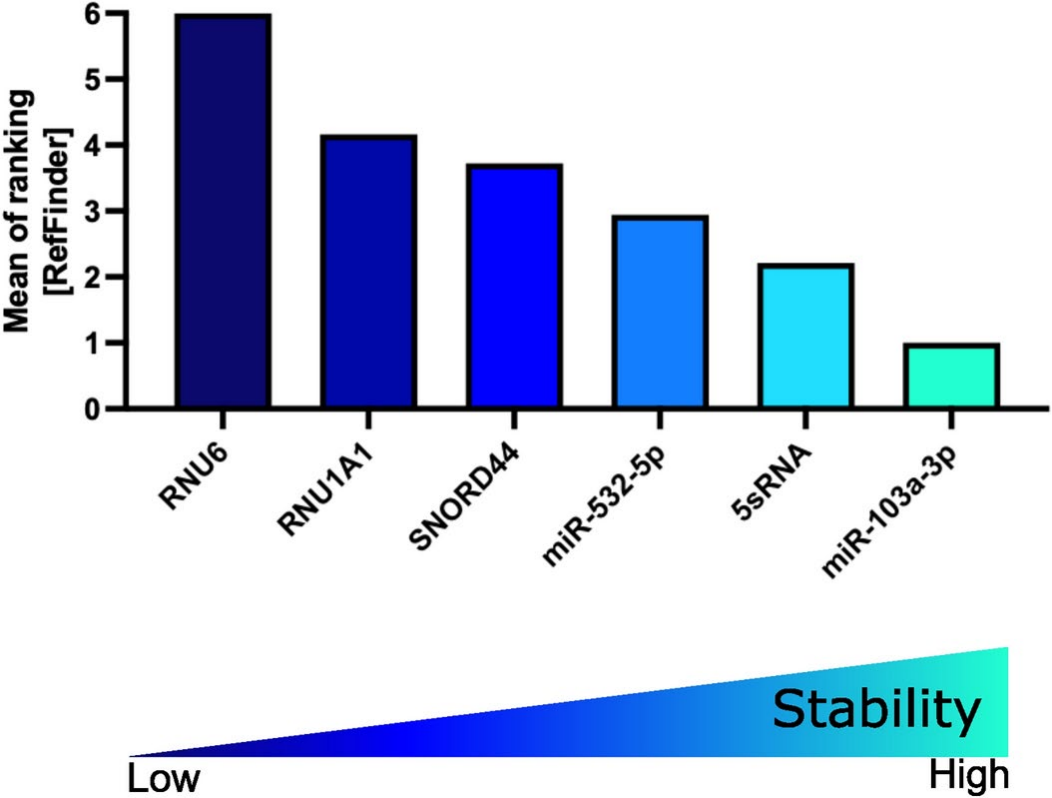
RefFinder on PDX ALL samples of the validation cohort. Mean of ranking according to the Normfinder, BestKeeper, geNorm, and ∆CT method applying the RefFinder algorithm. Decreasing values indicate increasing stability. PDX: patient-derived xenograft; ALL: acute lymphoblastic leukemia.

The CT values in the validation cohort ranged from 11.24 for 5sRNA to 31.93 for miR-532-5p (Supplementary Fig. 2 and Supplementary Table 3). Applying the Normfinder (Supplementary Fig. 3A), BestKeeper (Supplementary Fig. 3B), or ∆CT algorithm (Supplementary Fig. 3C-D and Supplementary Table 4) to the validation cohort identified miR-103a-3p as the most reliable miRNA reference. When we grouped the samples according to the site of infiltration (spleen, BM, CNS), the stability of miR-103a-3p remained high ranking second with 5sRNA ranking first (Supplementary Fig. 3E). Interestingly, although performing worse in the previous algorithms, miR-532-5p in combination with miR-103a-3p was identified as the best combination according to the geNorm algorithm (Supplementary Fig. 3F). Moreover, RefFinder identified miR-103a-3p as the most reliable miRNA, with miR-532-5p ranking third (Fig. 10 and Table 7).

**Table 7:** RefFinder ranking of PDX ALL samples of the validation cohort.

miRNA reference	Rank				
	RefFinder	Normfinder [Stability Value]	BestKeeper [STD]	geNorm [Stability Value]	∆CT [Means of STD]
miR-103a-3p	1	0.926	0.757	0.908^★^	1.601
5sRNA	2	1.025	0.777	1.174	1.694
miR-532-5p	3	1.643	0.833	0.908^★^	1.961
SNORD44	4	1.222	1.364	1.502	1.83
RNU1A1	5	1.232	1.786	1.692	1.783
RNU6	6	1.819	2.319	1.828	2.101

*Best performance in data normalization using miR-103a-3p and miR-532-5p in combination according to geNorm algorithm. PDX: patient-derived xenograft; ALL: acute lymphoblastic leukemia.

**Fig. 11:**
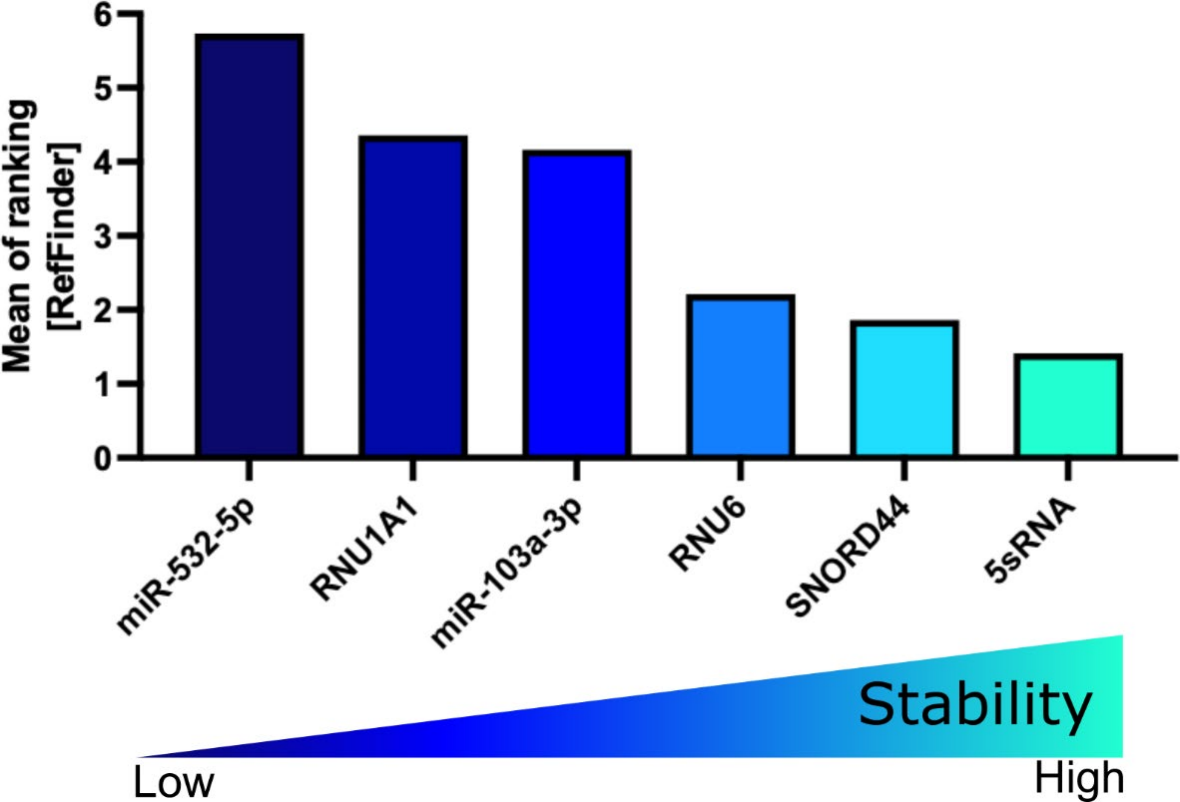
RefFinder on healthy controls of the validation cohort. Mean of ranking according to the Normfinder, BestKeeper, geNorm, and ∆CT method applying the RefFinder algorithm. Decreasing values indicate increasing stability.

To assess the influence of sample type on reference gene expression, we assessed miRNA expression levels in six additional PBMCs of HCs (3 female and 3 male). The mean CT values ranged from 12.7 for 5sRNA to 29.69 for miR-532-5p (Supplementary Table 5) with heterogenous expression of the miRNA references in the validation cohort of HCs (Supplementary Fig. 4A). SNORD44 was identified as most stably expressed when either the Normfinder (Supplementary Fig. 4B) or BestKeeper algorithm (Supplementary Fig. 4C) were used. Applying the ∆CT (Supplementary Fig. 4D, E and Supplementary Table 6) or geNorm algorithm identified 5sRNA and 5sRNA/RNU6 to be used as reliable references, respectively (Supplementary Fig. 4F). Despite 5sRNA ranking first in the RefFinder algorithm, SNORD44 still demonstrated stable expression, validating its reliability as a reference gene in PBMCs of HCs (Fig. 11).

Applying the RefFinder algorithm to the combined PDX ALL samples and HC specimens of the validation cohort identified miR-103a-3p as most stably expressed (Supplementary Fig. 5A, Supplementary Table 7). Importantly, also in the validation cohort, some miRNA references are characterized by increased score variations in ALL samples compared to HCs (Supplementary Fig. 5B). However, assessing intrinsic sample type differences by applying grouping to the Normfinder algorithm (ALL samples and HCs) still identified miR-103a-3p as the most stable reference in the combined cohort (Supplementary Fig. 5C).

### Influence of reference gene selection on miRNA expression in ALL

Selecting stably expressed miRNA references is critical for accurately identifying true biological differences in miRNA expression levels^13^. Therefore, we aimed at evaluating the consequences of choosing stably or varying expressed miRNA references on the expression data of miRNAs in PDX ALL samples. We assessed the expression of miR-181a-5p, which was shown to be consistently overexpressed in ALL patients as compared to healthy controls^14^ in BM ALL PDX samples in comparison to PBMCs of HCs. First, we aimed at demonstrating the applicability and relevance of xenograft-derived data in reflecting patient-specific features in terms of miRNA expression levels. Therefore, we analyzed the expression of miRNA references in a set of primary patient samples, which were used to establish the mouse model. Notably, we identified a significant positive correlation between patient- and xenograft-derived miRNA expression levels (Supplementary Fig. 6 and Supplementary Table 8). Next, we assessed the expression of miR-181a-5p in the identification cohort (Supplementary Table 1) in relation to miR-532-5p (most stably expressed in ALL samples; Fig. 6), miR-532-5p/miR-103a-3p (combining the most stable miRNA references in ALL and HCs specimens; Fig. 9A), or SNORD44 (most stably expressed in HCs; Fig. 8).

**Fig. 12:**
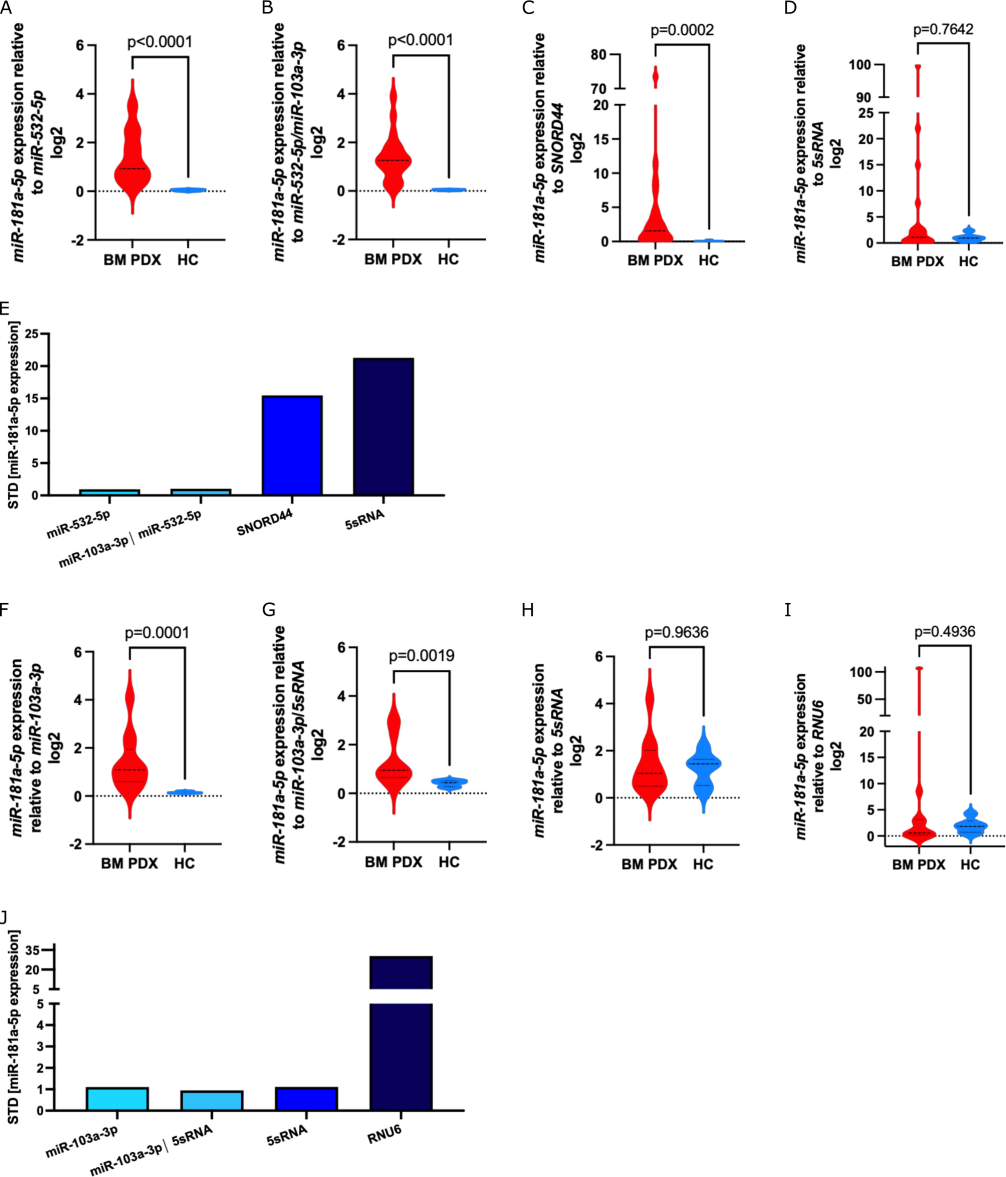
Influence of miRNA reference on analyzed miR-181a-5p expression in BM PDX ALL specimens. The expression of miR-181a-5p in PDX BM specimens as compared to HCs of the identification cohort was analyzed by normalizing the miRNA expression to miR-532-5p (**A**), miR-532-5p/miR-103a-3p (**B**), SNORD44 (**C**), and 5sRNA (**D**). Analysis of STD of miR-181a-5p expression dependent on miRNA reference used for data normalization in the identification cohort (**E**). MiRNA-181a-5p expression in relation to miR-103a-3p (**F**), miR-103a-3p/5sRNA (**G**), 5sRNA (**H**), and RNU6 (**I**) was assessed in BM-derived ALL cells compared to HCs of the validation cohort. STD of miR-181a-5p expression in association with used reference in the validation cohort (**J**). Mann–Whitney U test. BM: bone marrow; PDX: patient-derived xenograft; ALL: acute lymphoblastic leukemia; STD: standard deviation.

As shown in Fig. 12A-C, miR-181a-5p is significantly upregulated in BM-derived ALL samples as compared to HCs independent of the miRNA reference used. However, when using 5sRNA, the worst performing miRNA reference in the cohort of ALL and HCs (Fig. 9A), no significant increase was observed in miR-181a-5p expression in BM PDX specimens as compared to HCs (Fig. 12D). The effect of using distinct references with varying stability was also reflected in the STD of miR-181a-5p expression in BM PDX ALL samples ranging from 0.9173, to 1.029, 15.46, and 21.28 for miR-532-5p, the combination miR-532-5p/miR-103a-3p, SNORD44, or 5sRNA, respectively (Fig. 12E).

In line with these analyses, we evaluated the expression of miR-181a-5p in BM-derived ALL cells as compared to PBMCs from HCs in the validation cohort (Supplementary Table 3). MiR-181a-5p expression levels were normalized to miR-103a-3p (most stably expressed in ALL samples; Fig. 10), miR-103a-3p/5sRNA (most stable references in ALL and HCs; Supplementary Fig. 5A), 5sRNA (most stable reference in PBMCs derived from HCs; Fig. 11), and to RNU6 (worst performing reference; Supplementary Fig. 5A). While increased expression levels of miR-181a-5p were observed in BM-derived PDX ALL samples when normalized to miR-103a-3p (Fig. 12F) or the combination of miR-103a-3p/5sRNA (Fig. 12G), normalization using 5sRNA (Fig. 12H) or RNU6 (Fig. 12I) did not reveal significantly altered miR-181a-5p levels. The STD of miR-181a-5p expression ranged from 1.102 to 0.9512, 1.114, and 30.38 when normalized to miR-103a-3p, miR-103a-3p/5sRNA, 5sRNA or RNU6, respectively (Fig. 12J).

## Discussion

Understanding the molecular biology that gives rise to ALL is the basis for developing novel treatment modalities. Recent advances have been made in analyzing the role of miRNAs in leukemogenesis, response to therapy, and patient outcome, substantiating that the identification of novel oncogenic or tumor-suppressive ALL-associated miRNAs can be useful for the development of novel therapies^7^. MiRNA expression can be detected using various methods, including microarrays and qPCR with miRNA-specific primers or probes^32^. While microarrays enable high-throughput analysis, qPCR is faster, simpler, and more sensitive. Regardless of the method, normalization is crucial to reduce technical variation and ensure accurate data interpretation^13^. Normalization can be absolute or relative^32^. Absolute normalization uses standard curves but doesn’t account for variations in RNA quality. In contrast, relative quantification relies on stable exogenous or endogenous references, with endogenous controls adjusting for sample variability, making them a more reliable option^54^. Tools, including those used in this study, help identify the most stable references for accurate normalization.

**Fig. 13:**
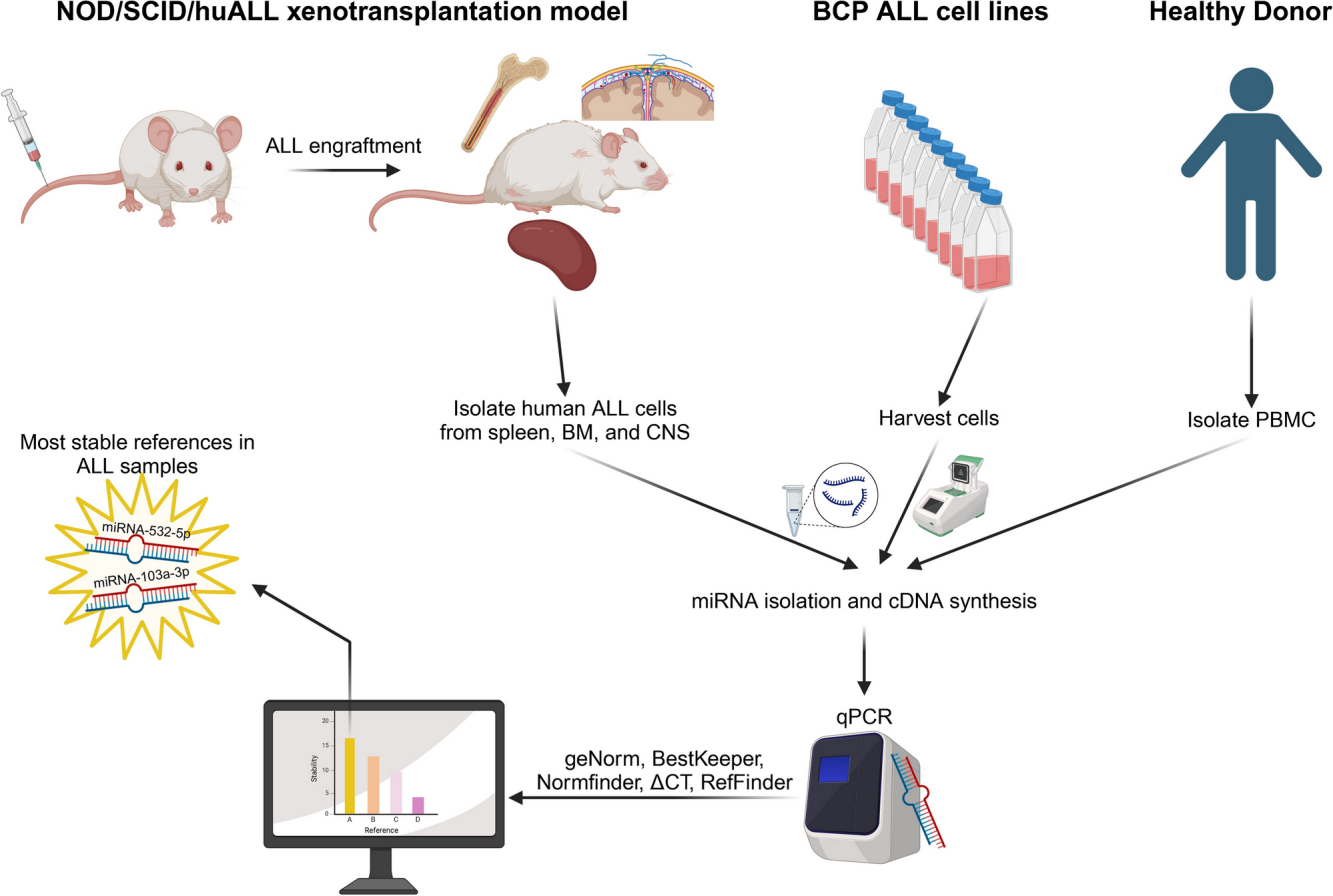
Graphical summary. After establishing the xenograft mouse model, human leukemia cells from spleen, BM, and CNS were isolated and, together with cells from nine individual BCP ALL cell lines (NALM-6, REH, RS4;11, KOPN-8, UoCB6, RCH-ACV, MHH-CALL2, EU3, and HAL-01) and PBMCs derived from six human healthy donors, were subjected to miRNA isolation and cDNA synthesis. The expression levels of six standards (5sRNA, SNORD44, RNU6, RNU1A1, miR-103a-3p, and miR-532-5p) in all samples were analyzed by qPCR. Four different algorithms (geNorm, BestKeeper, Normfinder, ΔCT) were applied, identifying miR-103a-3p and miR-532-5p as the most reliable references to be used in accurate qPCR normalization in miRNAs studies in BCP ALL xenografts. NOD/SCID/huALL: non-obese diabetic/severe combined immunodeficiency/human acute lymphoblastic leukemia; BM: bone marrow; CNS: central nervous system; PBMC: peripheral blood mononuclear cell. Created in BioRender. Meyer, L. (2024) https://BioRender.com/z55z209

Here, we evaluated the expression of six endogenous references in BCP ALL specimens using qPCR and assessed their stability by applying four different algorithms. We show miR-103a-3p and miR-532-5p to be stably expressed and to be used for miRNA qPCR data normalization in ALL cell lines and PDX ALL samples derived from BM, spleen, and CNS (Fig. 13), enabling accurate analysis of miRNA expression levels in different organ compartments commonly infiltrated in ALL patients. Interestingly, a recent study analyzed the expression levels of 47 candidate miRNAs (including miR-532-5p and miR-103a-3p) in extracellular vesicles derived from cerebrospinal fluid of ALL patients and platelet-free serum from peripheral blood of healthy individuals. While miR-103a-3p was identified to be differentially expressed in extracellular vesicles derived from the cerebrospinal fluid of patients with and without CNS involvement, miR-532-5p was identified as most stably expressed and was thus used as the reference^23^. However, the expression stability of miR-532-5p and miR-103a-3p in BCP ALL cells obtained from BM, CNS, or peripheral blood of patients was not assessed in this study. In contrast, our study is the first to demonstrate that miR-532-5p in combination with miR-103a-3p is stably expressed in leukemia cells derived from BCP ALL cell lines and PDX samples obtained from different organ compartments.

By now, a variety of different miRNA references, such as miR-16 or RNU6, have been used in ALL studies evaluating miRNA expression levels^14,35^. However, we detected varying expression levels of frequently used miRNA references in our ALL samples, which was further substantiated by a differential stability ranking of these miRNAs when the control group of healthy individuals was included in the ALL cohort. In line, using miR-16 for miRNA data normalization^55–57^ should be considered cautiously as it was shown to be differentially expressed in ALL patients compared to healthy controls^58–60^. Similarly, RNU6 was detected as differentially expressed in breast carcinoma cells as compared to HCs^61^, thus highlighting the importance of evaluating the expression stability of miRNA references before being used in miRNA studies.

Varying expression levels of frequently used miRNA references also differ between healthy and diseased tissues. Although SNORD44 was identified as stably expressed in PBMCs derived from HCs in both the identification and validation cohort, the stability of miR-103a-3p and miR-532-5p remained high when analyzed in combination with ALL specimens. However, we recognize the potential bias due to the disproportionate sample sizes, as the smaller HC cohort may be overshadowed by the larger ALL cohort, potentially leading to an overrepresentation of stability scores from ALL specimens. Despite this, the increased ranking of miR-103a-3p was only observed in the identification cohort, as reflected in Normfinder grouping results, where RNU6 was identified as the most stable reference when intra-group variation was considered. In contrast, in the validation cohort, miR-103a-3p remained stable even upon Normfinder grouping, which is further supported by the low variation in ranking scores in PDX ALL samples across both cohorts.

In addition to their varying expression in pathological conditions, miRNAs are also differentially expressed across distinct cell types and cell populations^61,62^. The expression of miR-532-5p was described to be increased in monocytes and plasmacytoid dendritic cells but not in other immune cell subsets^62^. This is further substantiated by the observation that RNU6 expression levels differ in cancerous epithelial and mesenchymal cells^61^. Hence, we took advantage of the NOD/SCID/huALL xenotransplantation model, allowing us to analyze the expression of miRNA references in leukemia cells derived from different organ compartments. Importantly, the previously observed engraftment phenotype of different PDX samples presenting with high or minor CNS manifestation in the NOD/SCID mouse model^17^ was also observed within the ALL cohorts analyzed here. Interestingly, we also identified varying expression levels of frequently used miRNA references, pointing to a diverse expression in ALL specimens of different organ compartments. However, when applying grouping to the Normfinder algorithm to both the identification and validation cohort, the stability of miR-103a-3p (in both cohorts) and miR-532-5p (in the identification cohort) was maintained high, substantiating the reliability of using these miRNAs for data normalization in miRNA studies on ALL specimens. GeNorm further strengthens these findings in both cohorts, confirming miR-103a-3p in combination with miR-532-5p as most stable references in ALL specimens. Importantly, geNorm uses pairwise comparisons to assess gene stability across all samples, identifying reference genes with minimal variation, even across different experimental conditions or biological variability, making this algorithm a robust method for selecting stable reference genes in heterogeneous cohorts, including ALL.

Besides organ- and tissue-specific expression patterns, miRNA profiles have also been used to identify ALL subgroups: B- and T-cell ALL patients can be distinguished according to miRNA expression levels^63–65^, and the cytogenetic BCP ALL subgroups of *KMT2A-*, *ETV6::RUNX1-*, *PBX1-*rearranged and hypodiploid cases were shown to cluster according to their miRNA profiles^25^. The number of samples used in this study does not allow us to draw conclusions about the stability of reference genes across different BCP ALL subgroups. However, we show that expression levels of miRNA references correlate between primary material and PDX samples derived from BM, indicating that, as in a previous study^9^, miRNA expression data obtained from xenografts is likely applicable to patient samples.

The profound effect of using miRNA references with varying expression stabilities was demonstrated when we used the least stable miRNA reference 5sRNA (identification cohort) and RNU6 (validation cohort) for data normalization analyzing the expression of miR-181a-5p in PDX BM as compared to HCs: Using inappropriate references with only minor expression stability resulted in a loss of statistical significance and, hence, diminishing the biological relevance of these findings. The importance of using stable miRNA references for data normalization extends beyond our findings. Inconsistent or unstable references can lead to skewed results, masking biologically relevant differences or producing false positives. This is in line with a previous study on human cardiac tissue, which analyzed the effect of miRNA references on the outcome of differential miRNA expression, showing that in case of small expressional differences, the use of a stable miRNA reference is of tremendous importance to detect significant miRNA expression changes^66^.

Several limitations should be considered when interpreting the findings of this study. First, we analyzed a relatively small sample size, particularly for the HC cohorts, and no patient-derived material was used in this study; instead, patient-derived xenograft models and cell lines were employed. While PDX models are valuable for mimicking patient biology^18–20^, they may not fully capture the complexities of primary human samples. However, our study allowed us to analyze expression levels across different organ compartments, which is often highly limited when working with patient-derived material. The study design also focused on validating a limited set of miRNA references rather than a broader, high-throughput array approach. This may have restricted the scope of miRNA profiling and prevented the discovery of other potentially stable miRNAs. Furthermore, laboratory-related factors, such as sample preparation and handling, likely influenced the results, as evidenced by the variation in CT values between the identification and validation cohorts. Such discrepancies suggest that technical factors could contribute to the differences in miRNA stability across cohorts. Additionally, the study did not account for miRNAs associated to the heterogeneity of ALL subtypes^24,25^. The inability to analyze miRNA stability in a subtype-specific manner is a significant limitation, as it prevents the identification of potential reference miRNAs that could be more suitable for specific ALL subtypes.

In conclusion, we provide the first study analyzing the stability of a variety of miRNA references in ALL cell lines and PDX specimens derived from different organ compartments and identifying miR-103a-3p and miR-532-5p as the most stably expressed reference miRNAs in BCP ALL models. Since patient-derived material from different organ compartments is exceedingly rare and difficult to obtain, our work with PDX ALL samples provides essential groundwork for future studies focusing on miRNAs as diagnostic or prognostic markers. Given the significant differences in miRNA reference expression stability between ALL specimens and HCs and expressional variations between cohorts, we suggest evaluating the stability of standards within the sample cohort to avoid introducing bias due to varying reference levels.

## Methods

### Cell lines and cell culture

The ALL cell lines NALM-6, REH, RS4;11, KOPN-8, RCH-ACV, MHH-CALL2, EU3, and HAL-01 were obtained from the German Collection of Microorganisms and Cell Cultures GmbH (DSMZ, Braunschweig, Germany). UoC-B6 cells were kindly provided by Dr. Rowley, Chicago, USA. Cell lines were regularly tested for Mycoplasm contamination (MycoAlert® Mycoplasma Detection Kit, Lonza, Basel, Switzerland) and were authenticated by short-tandem repeat profiling (GenePrint® 10 System, 12 Promega Madison, Wisconsin, USA). Cells were cultured in RPMI-1640 supplemented with 20% fetal calf serum, 1% L-glutamine, and 1% penicillin/streptomycin (Thermo Fisher Scientific, Waltham, NA, USA).

### PDX samples

Peripheral blood or BM of pediatric patients diagnosed with B-cell precursor ALL were obtained after informed consent was given by the patient and/or their legal guardian after approval and in accordance with the local ethical review board (Ethikkommission der Universität Ulm; No. 461/19). Animal experiments were approved by the appropriate authority (Tierschutz-Fachreferat 35, Regierungspräsidium Tübingen; No. V.048) and were performed in accordance with local guidelines and regulations, complying with the ARRIVE guidelines. 3–10 × 10^6^ leukemia cells were intravenously transplanted into NOD/SCID (NOD.Cg-Prkdcscid/J) mice. At the time of leukemia-related morbidity, mice were sacrificed, and leukemia cells engrafted in the spleen, BM, and CNS were isolated^9,17^. Leukemia loads in the different organ compartments were analyzed by flowcytometric stainings of cells using APC anti-human CD19 and PE anti-mouse CD45 antibodies (BD Biosciences, Franklin Lakes, NJ, USA), and viability of cells was analyzed according to forward and side scatter (FSC/SSC) criteria using the BD® LSR II Flow Cytometer (BD Biosciences, Franklin Lakes, NJ, USA).

### PBMCs from HCs

To analyze the expression of miRNA references and miR-181a-5p in peripheral blood mononuclear cells (PBMCs) of HCs, we obtained buffy coats from anonymous blood healthy donors from the Institute of Clinical Transfusion Medicine and Immunogenetics Ulm. Donors provided written informed consent to the blood being used for research purposes according to local regulations (Ethikkommission der Universtät Ulm).

### MLPA

PDX ALL samples were analyzed for the presence of genetic alterations performing SALSA MLPA analyses. We extracted DNA of leukemia cells derived from the spleen (QIAmp DNA Blood Kit, Qiagen, Hilden, Germany) and used MRC Holland probe mix P335 to assess copy number variations and single nucleotide polymorphisms according to the manufacturer’s instructions. We have denaturated 50–100 ng DNA containing 10 mM Tris–HCL pH 8–8.5 at 98 °C for 5 min. Subsequently, the probe mix was added and hybridized for 16 h at 60 °C. Following the manufacturer’s instructions, the ligation and PCR reactions were prepared. Capillary electrophoresis was performed using the GeneScan™ 500 ROX™ Dye Size Standard and was run on Applied Biosystems™ 3500xL Genetic Analyzer (ThermoFisher Scientific, Waltham, NA, USA). MLPA data were analyzed using Coffalyser.Net™ (version 24.0.1; https://www.mrcholland.com/technology/software/coffalyser-net; MRC Holland, Amsterdam, Netherlands).

### RNA extraction and cDNA synthesis

Total RNA was extracted using the *Quick*-RNA Miniprep Kit (Zymo Research, Freiburg, Germany), and concentration was determined at 260 nm using the NanoDrop 2000 (ThermoFisher Scientific, Waltham, NA, USA). Reverse transcription of miRNAs was performed on 20 ng RNA using the miRCURY LNA RT Kit (Qiagen, Hilden, Germany) following the manufacturer’s instructions.

### qPCR

Expression levels of miRNAs were assessed by qPCR with the miRCURY LNA SYBR Green PCR Kit and the miRCURY LNA miRNA PCR Assay (Qiagen, Venlo, Netherlands) using primers for RNU6 (YP00203907), RNU1A1 (YP00203909), SNORD44 (YP00203902), 5sRNA (YP00203906), miR-103a-3p (YP00204063), miR-532-5p (YP00204221), and miR-181a-5p (YP00206081).

### Statistics and Software

The 2^-∆∆CT^ method was used to analyze miRNA expression profiles in relation to references^67^. Prism 10 (version 10.3.1; https://www.graphpad.com/; GraphPad software, Boston, MA, USA) was used for graphical illustration and statistical analyses. A p-value p < 0.05 was considered significant. Expressional stability applying the ∆CT algorithm was analyzed according to reference^29^. The online free-available tools Normfinder^28^ (https://www.moma.dk/software/normfinder), geNorm^26^ (https://genorm.cmgg.be/), BestKeeper^27^ (https://www.gene-quantification.de/bestkeeper.html), and RefFinder^53^ (https://www.ciidirsinaloa.com.mx/RefFinder-master/) were used to evaluate the stability of miRNA expression. Flow cytometry data were analyzed using FlowJo™ (version 10.4; https://www.flowjo.com/; Ashland, OR, USA).

## Supplementary Information

Supplementary Information.

## Data Availability

All data not provided in the manuscript and the supplementary information are available from the authors upon request.
