# Supplementary material for: Benchmarking miRNA reference genes in B-cell precursor acute lymphoblastic leukemia
Source: Sci Rep. 2024 Nov 2;14:26390. doi: 10.1038/s41598-024-77733-8 (PMC11531470; doi:10.1038/s41598-024-77733-8)
Supplement: Supplementary file 1 — Supplementary Information. [file 41598_2024_77733_MOESM1_ESM.pdf]

Benchmarking miRNA Reference Genes in B-Cell Precursor Acute Lymphoblastic Leukemia

Teresa Mack[1°], Tommaso Gianferri[1°], Alexandra Niedermayer[1,2], Klaus-Michael Debatin[1], Lüder H. Meyer[1†], and Vera Muench[1†*]

[1] Department of Pediatrics and Adolescent Medicine, Ulm University Medical Center, Ulm, Germany
[2] International Graduate School in Molecular Medicine, Ulm University, Ulm, Germany
°Authors contributed equally to the work
†Authors contributed equally to the work
*Corresponding author
vera.muench@uniklinik-ulm.de

Supplementary Table 1 CT values of PDX ALL samples and ALL cell lines of the identification cohort.

		RNU6	miR-103a-3p	5sRNA	RNU1A1	SNORD44	miR-532-5p
Cell lines	KOPN-8	24.35	23.72	17.88	20.25	21.45	28.88
	UoCB6	24.62	23.63	17.82	20.41	21.24	28.75
	EU3	24.86	23.13	18.31	20.47	20.91	28.12
	NALM-6	23.32	23.57	16.06	18.01	20.96	27.9
	RS4;11	23.61	22.34	18.3	20.39	21.33	29.53
	RCH-ACV	23.75	22.62	17.51	18.92	21.19	29.1
	MHH-CALL2	22.93	23.86	17.27	18.92	21.34	29.63
	HAL01	22.81	22.75	17.07	20.02	22.53	28.88
	REH	23.8	22.62	16.69	19.55	21.13	30.53
Spleen	X001	24.7	24.53	18.37	21.18	22.68	29.18
	X002	24.24	22.18	18.15	19.99	21.9	27
	X003	24.84	23.68	13.77	19.4	23.38	30.17
	X004	30.51	25.09	11.95	21.6	26.62	30.12
	X005	20.75	23.1	12	15.95	21.53	28.46
	X006	21.58	23.29	15.85	19.37	20.31	29.74
	X007	21.35	23.61	15.54	17.53	20.61	28.47
	X008	20.86	24.23	20.4	16.62	21.19	28.1
	X009	26.23	22.09	18.61	22.77	23.16	28.42
	X010	26.06	23.63	14.06	18.22	23.75	26.84
	X011	17.86	22.2	11.78	14.04	19.42	29.19
	X012	26.61	25.3	21.72	23.68	24.36	30.35
	X013	25.14	23.67	11.99	19.13	24.62	28.75
	X014	22.71	23.26	14.72	18.93	22.19	28.78
	X015	26.06	23.19	12.93	19.23	24.99	27.94
	X016	21.85	23.69	14.09	17.64	21.07	29.11
	X017	23.44	25.48	15.26	18.96	21.2	29.54
	X018	23.76	25.35	17.54	20.3	21.94	30.29
	X019	19.84	23.16	14.02	15.62	22.99	26.13
	X020	23.62	24.82	11.81	18.34	24.48	29.5
	X021	23.03	24.54	14.05	18.61	20.98	28.34
	X022	24.37	23.55	17.45	21.22	20.93	28.11
BM	X001	25.99	24.15	15.84	20.88	22.43	28.84
	X002	24.91	22.13	19.17	20.67	22.33	27.56
	X003	28.81	24.09	12.76	18.7	24.58	29.66
	X004	28.4	26.28	11.34	19.98	27.4	31.23
	X005	24.72	24.76	10.64	16.93	26.57	30.14
	X006	19.69	24.18	16.87	19.93	18.95	31.22
	X007	24.72	22.95	14.75	18.99	23.12	28.43
	X008	23.74	18.41	18.07	20.52	21.56	27.75

	**X009**	26.35	22.5	20.34	22.65	22.93	28.46
	**X010**	26	23.04	12.07	16.42	27.42	27.66
	**X011**	27.49	24.11	14.63	17.97	22.05	29.83
	**X012**	22.96	25.99	21.48	25.27	26.68	31.3
	**X013**	26.15	24.47	14.24	18.5	26.34	29.16
	**X014**	23.86	23.67	12.54	19.13	24.78	29.93
	**X015**	24.17	23.2	14.84	18.69	25.65	27.93
	**X016**	25.6	23.44	11.05	19.19	23.89	28.86
	**X017**	21.74	25.82	17.33	20.12	20.44	30.1
	**X018**	23.23	25.45	17.72	17.86	21.93	29.57
	**X019**	22.37	21.96	15.19	17.68	21.63	26.1
	**X020**	27.22	26.79	11.48	19.74	31.19	31.55
	**X021**	21.53	24.06	15.69	19.27	20.31	27.89
	**X022**	27.46	26.02	16.82	24.97	26.64	29.18
**CNS**	**X001**	25.13	23.8	17.64	20.89	21.8	28.72
	**X002**	24.54	22.53	19.1	21	21.65	27.37
	**X003**	23.86	24.15	13.18	17.75	21.95	30.26
	**X004**	26.57	26.11	12.77	19.64	24.3	29.77
	**X005**	23.79	23.75	15.46	19.52	21.48	29.22
	**X006**	19.8	23.65	15.71	18.51	21.13	29.53
	**X007**	22.22	22.97	16.89	18.6	21.05	28.57
	**X008**	23.74	23.09	17.65	18.22	22.36	27.7
	**X009**	26.44	22.98	18.56	22.81	22.71	29.33
	**X010**	24.44	22.87	16.25	19.14	22.09	26.62
	**X011**	22.39	23.53	15.59	17.77	20.82	29.84
	**X012**	26.41	25.61	23.67	25.64	24.93	30.48
**Mean CT values**		**24.15**	**23.76**	**15.85**	**19.52**	**22.79**	**28.98**

PDX: patient-derived xenograft; ALL: acute lymphoblastic leukemia; BM: bone marrow; CNS: central nervous system.

Supplementary Table 2 CT values of PBMCs from healthy controls of the identification cohort.

		RNU6	miR-103a-3p	5sRNA	RNU1A1	SNORD44	miR-532-5p
Healthy Controls	HC1	19.47	20.8	13.95	15.95	19.27	25.79
	HC2	22.61	20.32	16.6	19.18	19.69	26.79
	HC3	19.05	19.62	14.13	16.82	18.5	25.21
	HC4	21.96	18.84	15.63	18.67	18.63	24.06
	HC5	21.97	23.85	18.94	15.88	20.07	23.49
	HC6	23.61	19.97	15.22	16.56	21.73	24.36
**Mean CT value**		**21.45**	**20.57**	**15.75**	**17.18**	**19.65**	**24.95**

PBMC: peripheral blood mononuclear cells; HC: healthy control.

Supplementary Table 3 CT values of PDX ALL samples of the validation cohort.

		RNU6	miR-103a-3p	5sRNA	RNU1A1	SNORD44	miR-532-5p
Spleen	Validation X001	15.33	22.00	10.59	12.66	19.85	32.22
	Validation X002	17.27	24.56	10.30	12.84	20.93	33.26
	Validation X003	16.45	22.18	10.34	12.26	21.75	31.29
	Validation X004	15.70	22.97	10.19	11.89	20.14	30.62
	Validation X005	16.56	22.91	10.58	12.87	21.80	31.96
	Validation X006	17.78	24.04	11.40	14.36	22.93	32.45
	Validation X007	15.52	22.17	10.98	11.91	20.79	29.85
	Validation X008	18.60	24.83	11.01	13.83	23.70	33.13
	Validation X009	17.89	23.71	11.52	14.06	23.27	30.44
	Validation X010	15.46	23.96	10.63	12.17	20.48	34.55
	Validation X011	21.35	23.34	12.57	16.59	23.06	30.69
	Validation X012	24.05	24.50	12.89	19.12	23.96	32.51
BM	Validation X001	14.66	22.20	10.13	11.56	20.04	32.78
	Validation X002	16.72	23.78	10.26	12.19	21.01	31.72
	Validation X003	19.59	23.49	10.58	14.53	24.11	32.53
	Validation X004	17.71	24.29	10.82	13.55	22.64	32.73
	Validation X006	17.17	24.48	11.91	14.09	22.71	32.70
	Validation X007	14.64	21.43	10.19	10.76	19.74	29.85
	Validation X008	18.16	24.01	11.63	14.18	22.10	32.11
	Validation X009	22.22	25.08	10.97	16.68	29.12	32.04
	Validation X010	16.58	24.11	11.34	13.27	21.48	32.19
	Validation X011	19.73	22.68	12.08	14.22	22.22	30.66
	Validation X012	23.73	24.47	12.84	19.37	23.14	32.67
	Validation X013	23.70	24.65	10.69	16.66	25.69	32.49

CNS	**Validation X004**	15.63	24.15	10.37	12.54	20.59	32.14
	**Validation X006**	17.03	23.87	12.12	14.05	22.18	31.83
	**Validation X007**	15.65	22.48	11.25	12.68	20.78	31.25
	**Validation X008**	16.65	23.94	10.71	12.33	21.64	32.71
	**Validation X011**	20.42	23.47	12.61	17.66	22.46	30.88
	**Validation X012**	21.87	23.84	14.01	18.75	22.11	32.38
	**Validation X013**	21.16	23.62	10.93	15.59	23.79	31.15
**Mean CT values**		**18.23**	**23.59**	**11.24**	**14.17**	**22.27**	**31.93**

PDX: patient-derived xenograft; ALL: acute lymphoblastic leukemia; BM: bone marrow; CNS: central nervous system.

Supplementary Table 4 Mean of ∆CT values and STD of PDX ALL samples of the validation cohort.

	Mean	STD	Mean STD
RNU6 vs miR-103a-3p	-5.36	2.43	
RNU6 vs 5sRNA	6.99	2.32	
RNU6 vs RNU1A1	4.06	1.01	
RNU6 vs SNORD44	-4.04	1.83	
RNU6 vs miR-532-5p	-13.70	2.92	2.10
miR-103a-3p vs RNU6	5.36	2.43	
miR-103a-3p vs 5sRNA	12.35	1.15	
miR-103a-3p vs RNU1A1	9.42	2.00	
miR-103a-3p vs SNORD44	1.32	1.52	
miR-103a-3p vs miR-532-5p	-8.34	0.91	1.60
5sRNA vs RNU6	-6.99	2.32	
5sRNA vs miR-103a-3p	-12.35	1.15	
5sRNA vs RNU1A1	-2.93	1.64	
5sRNA vs SNORD44	-11.03	1.90	
5sRNA vs miR-532-5p	-20.69	1.47	1.69
RNU1A1 vs RNU6	-4.06	1.01	
RNU1A1 vs miR-103a-3p	-9.42	2.00	
RNU1A1 vs 5sRNA	2.93	1.64	
RNU1A1 vs SNORD44	-8.10	1.83	
RNU1A1 vs miR-532-5p	-17.76	2.44	1.78
SNORD44 vs RNU6	4.04	1.83	
SNORD44 vs miR-103a-3p	-1.32	1.52	
SNORD44 vs 5sRNA	11.03	1.90	
SNORD44 vs RNU1A1	8.10	1.83	
SNORD44 vs miR-532-5p	-9.66	2.08	1.83
miR-532-5p vs RNU6	13.70	2.92	
miR-532-5p vs miR-103a-3p	8.34	0.91	
miR-532-5p vs 5sRNA	20.69	1.47	
miR-532-5p vs RNU1A1	17.76	2.44	
miR-532-5p vs SNORD44	9.66	2.08	1.96

PDX: patient-derived xenograft; ALL: acute lymphoblastic leukemia; STD: standard deviation.

Supplementary Table 5 CT values of PBMCs from healthy controls of the validation cohort.

		RNU6	miR-103a-3p	5sRNA	RNU1A1	SNORD44	miR-532-5p
Healthy Controls	Validation HC1	19.62	22.49	12.84	16.27	19.11	29.46
	Validation HC2	21.08	23.04	13.79	17.35	20.58	29.90
	Validation HC3	19.14	24.07	11.84	14.67	19.84	31.80
	Validation HC4	18.72	22.21	11.81	14.56	19.69	29.45
	Validation HC5	20.83	21.89	13.30	16.91	19.81	28.73
	Validation HC6	19.58	21.63	12.61	15.36	19.82	28.79
**Mean CT value**		**19.83**	**22.56**	**12.70**	**15.85**	**19.81**	**29.69**

PBMC: peripheral blood mononuclear cells; HC: healthy control.

Supplementary Table 6 Mean of △CT values and STD of PBMCs derived from HCs of the validation cohort.

	Mean	Std. Deviation	Mean STD
RNU6 vs miR-103a-3p	-2.728	1.36	
RNU6 vs 5sRNA	7.13	0.2867	
RNU6 vs RNU1A1	3.98	0.3964	
RNU6 vs SNORD44	0.02167	0.7805	
RNU6 vs miR-532-5p	-9.857	1.668	0.90
miR-103a-3p vs RNU6	2.728	1.36	
miR-103a-3p vs 5sRNA	9.857	1.315	
miR-103a-3p vs RNU1A1	6.708	1.586	
miR-103a-3p vs SNORD44	2.748	0.9001	
miR-103a-3p vs miR-532-5p	-7.13	0.3314	1.10
5sRNA vs RNU6	-7.13	0.2867	
5sRNA vs miR-103a-3p	-9.857	1.315	
5sRNA vs RNU1A1	-3.152	0.4164	
5sRNA vs SNORD44	-7.108	0.7169	
5sRNA vs miR-532-5p	-16.99	1.629	0.87
RNU1A1 vs RNU6	-3.98	0.3964	
RNU1A1 vs miR-103a-3p	-6.708	1.586	
RNU1A1 vs 5sRNA	3.152	0.4164	
RNU1A1 vs SNORD44	-3.957	1.093	
RNU1A1 vs miR-532-5p	-13.84	1.91	1.08
SNORD44 vs RNU6	-0.02167	0.7805	
SNORD44 vs miR-103a-3p	-2.748	0.9001	
SNORD44 vs 5sRNA	7.108	0.7169	
SNORD44 vs RNU1A1	3.957	1.093	
SNORD44 vs miR-532-5p	-9.882	1.151	0.93
miR-532-5p vs RNU6	9.857	1.668	
miR-532-5p vs miR-103a-3p	7.13	0.3314	
miR-532-5p vs 5sRNA	16.99	1.629	
miR-532-5p vs RNU1A1	13.84	1.91	
miR-532-5p vs SNORD44	9.882	1.151	1.34

PBMC: peripheral blood mononuclear cells; HC: healthy control; STD: standard deviation.

Supplementary Table 7 RefFinder ranking of PDX ALL samples and HCs of the validation cohort.

	Rank				
**miRNA reference**	**RefFinder**	**Normfinder [Stability Value]**	**BestKeeper [STD]**	**geNorm [Stability Value]**	**∆CT [Means of STD]**
miR-103a-3p	1	0.917	0.848	0.952*	1.722
5sRNA	2	1.153	0.905	1.7	1.882
miR-532-5p	3	1.801	1.106	0.952*	2.152
SNORD44	4	1.491	1.484	1.473	2.067
RNU1A1	5	1.404	1.808	1.895	1.936
RNU6	6	1.824	2.224	1.99	2.18

*Best performance in data normalization using miR-103a-3p and miR-532-5p in combination according to the geNorm algorithm. PDX: patient-derived xenograft; ALL: acute lymphoblastic leukemia; HCs: healthy controls; STD: standard deviation.

Supplementary Table 8 CT values of primary patient material and corresponding BM-derived PDX ALL sample.

Patient	PDX	Patient	PDX	Patient	PDX	Patient	PDX
		miR-532-5p		miR103a-3p		RNU6	
P1	X002	31.94	27.56	23.89	22.13	17.61	24.91
P2	X005	36.89	30.14	26.87	24.76	20.13	24.72
P3	X006	36.29	31.22	27.18	24.18	19.33	19.69
P4	X007	36.71	28.43	29.88	22.95	20.72	24.72
P5	X008	31.32	27.75	23.67	18.41	20.53	23.74
P6	X009	34.60	28.46	25.34	22.50	21.61	26.35
P7	X011	31.76	29.83	22.91	24.11	18.25	27.49
P8	X013	31.49	29.16	23.28	24.47	18.49	26.15
P9	X015	31.24	27.93	23.64	23.20	18.29	24.17
P10	X016	37.57	28.86	26.92	23.44	21.19	25.6
P11	X018	35.50	29.57	25.81	25.45	20.07	23.23
P12	X020	29.32	31.55	22.23	26.79	16.21	27.22
P13	X021	28.92	27.89	22.26	24.06	18.42	21.53
P14	X022	31.28	29.18	25.79	26.02	27.18	27.46
P15	Validation X001	33.41	32.78	23.86	22.20	18.72	14.66
P16	Validation X002	31.04	31.72	23.35	23.78	17.06	16.72
P17	Validation X004	32.78	32.73	25.97	24.29	24.19	17.71
P18	Validation X006	30.66	32.70	22.82	24.48	18.55	17.17
P19	Validation X008	34.69	32.11	24.46	24.01	20.53	18.16
P20	Validation X010	31.40	32.19	23.86	24.11	20.68	16.58
P21	Validation X011	30.61	30.66	22.75	22.68	19.29	19.73
P22	Validation X012	32.82	32.67	23.47	24.47	16.27	23.73
P23	Validation X013	30.34	32.49	22.29	24.65	18.54	23.70

Patient	PDX	Patient	PDX	Patient	PDX	Patient	PDX
		5sRNA		RNU1A1		SNORD44	
P1	X002	12.23	19.17	14.09	20.67	21.67	22.33
P2	X005	16.46	10.64	18.59	16.93	20.75	26.57
P3	X006	14.59	16.87	15.85	19.93	20.04	18.95
P4	X007	16.50	14.75	18.13	18.99	21.49	23.12
P5	X008	12.48	18.07	17.28	20.52	22.15	21.56
P6	X009	15.55	20.34	19.22	22.65	22.72	22.93
P7	X011	11.66	14.63	13.85	17.97	19.92	22.05
P8	X013	13.22	14.24	15.45	18.5	20.81	26.34

P9	X015	12.61	14.84	14.58	18.69	20.83	25.65
P10	X016	17.49	11.05	19.84	19.19	20.41	23.89
P11	X018	18.44	17.72	17.55	17.86	20.99	21.93
P12	X020	10.58	11.48	13.27	19.74	18.46	31.19
P13	X021	13.87	15.69	17.57	19.27	19.56	20.31
P14	X022	11.83	16.82	19.52	24.97	26.48	26.64
P15	Validation X001	12.96	10.13	15.06	11.56	21.04	20.04
P16	Validation X002	11.72	10.26	13.77	12.19	19.42	21.01
P17	Validation X004	11.53	10.82	18.74	13.55	23.75	22.64
P18	Validation X006	12.21	11.91	14.94	14.09	20.58	22.71
P19	Validation X008	12.30	11.63	16.02	14.18	23.01	22.10
P20	Validation X010	13.04	11.34	16.99	13.27	22.12	21.48
P21	Validation X011	12.35	12.08	15.22	14.22	20.89	22.22
P22	Validation X012	9.91	12.84	11.69	19.37	19.92	23.14
P23	Validation X013	11.99	10.69	14.84	16.66	19.97	25.69

PDX: Patient-derived xenograft; ALL: acute lymphoblastic leukemia; BM: bone marrow.

Supplementary Figure 1

A

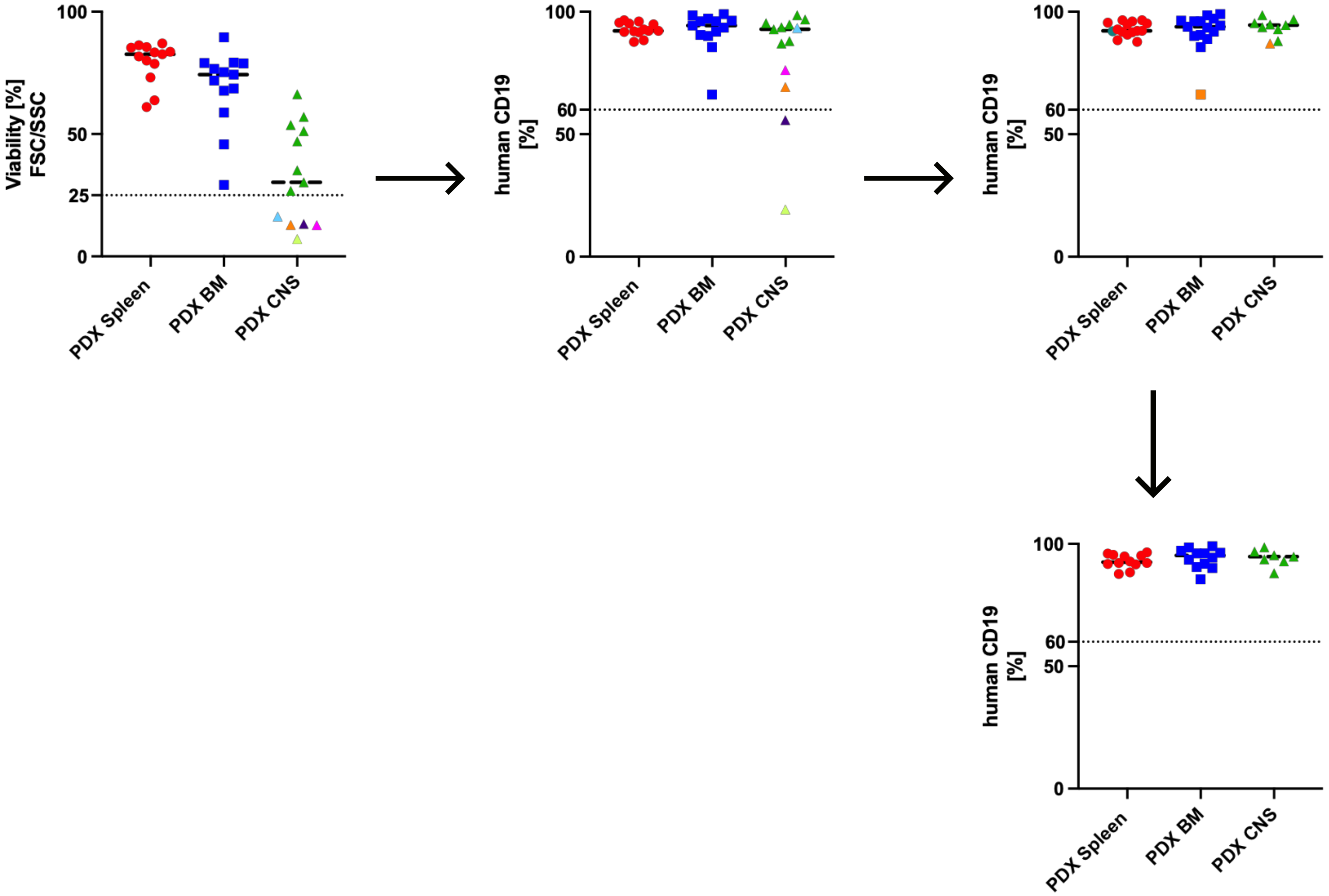

Supplementary Figure 1 PDX sample selection of the validation cohort. 13 PDX ALL samples (>25% viability and >60% human CD19; same coloring of excluded samples) were chosen for RNA extraction. One spleen- (green), BM- (brown), and CNS-derived (brown) sample each were excluded due to poor RNA quality (below right), leaving 12 BM-, and spleen-, and 7 CNS-derived ALL samples for further analyses. PDX: patient-derived xenograft; ALL: acute lymphoblastic leukemia; FSC/SSC: forward scatter/side scatter; BM: bone marrow; CNS: central nervous system.

Supplementary Figure 2

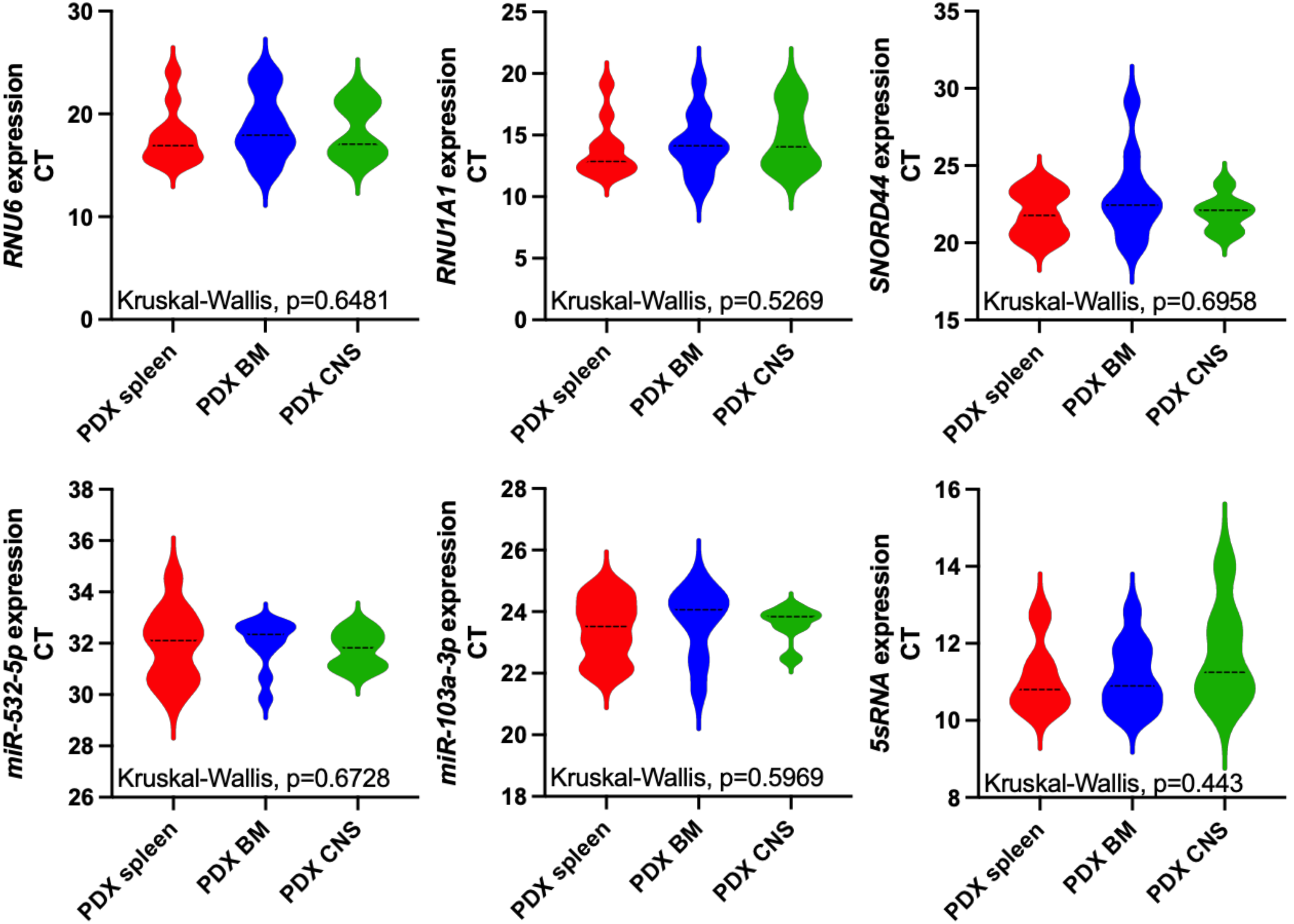

Supplementary Figure 2 Expression of miRNA references in PDX ALL specimens of the validation cohort. The expression of RNU6, RNU1A1, SNORD44, miR-532-5p, miR-103a-3p and 5sRNA were assessed in PDX ALL samples derived from spleen (n=12), BM (n=12), or CNS (n=7) of the validation cohort. The Kruskal-Wallis test was used to determine whether the median CT values of miRNA reference controls differ across ALL sample groups. PDX: patient-derived xenograft; ALL: acute lymphoblastic leukemia; BM: bone marrow; CNS: central nervous system.

Supplementary Figure 3

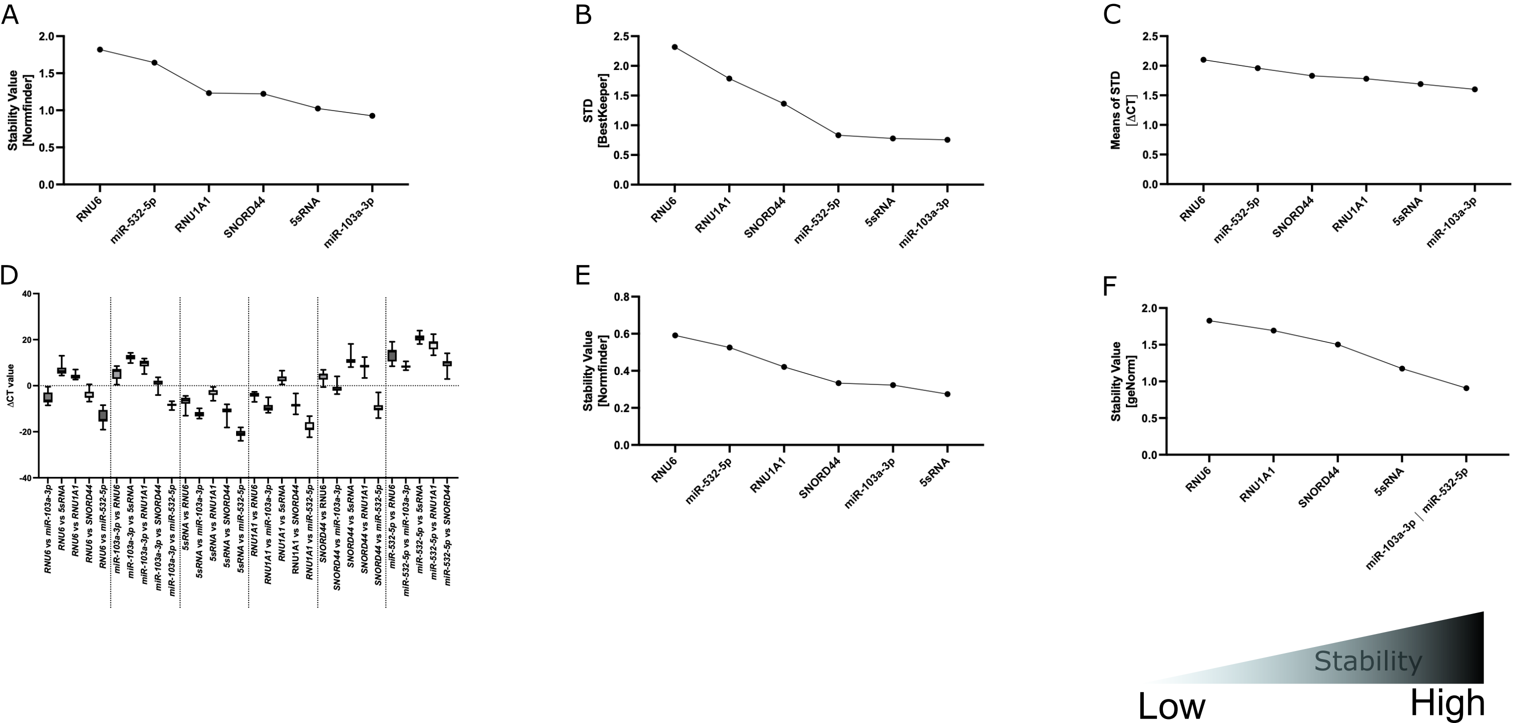

Supplementary Figure 3 Stability of miRNA references in PDX ALL samples of the validation cohort. The Normfinder (A; samples clustered in 1 group), BestKeeper (B), ∆CT (C and D), Normfinder applying grouping to the validation cohort (3 groups: spleen, BM, CNS; E), and geNorm algorithm (F) were used to assess reference stability within the validation cohort. For A-C, E, and F: decreasing values indicate increasing stability. E: ∆CT calculations based on CT[reference 1] – CT[reference 2]; mean CT values with min to max whiskers are shown. PDX: patient-derived xenograft; ALL: acute lymphoblastic leukemia; BM: bone marrow, CNS: central nervous system.

Supplementary Figure 4

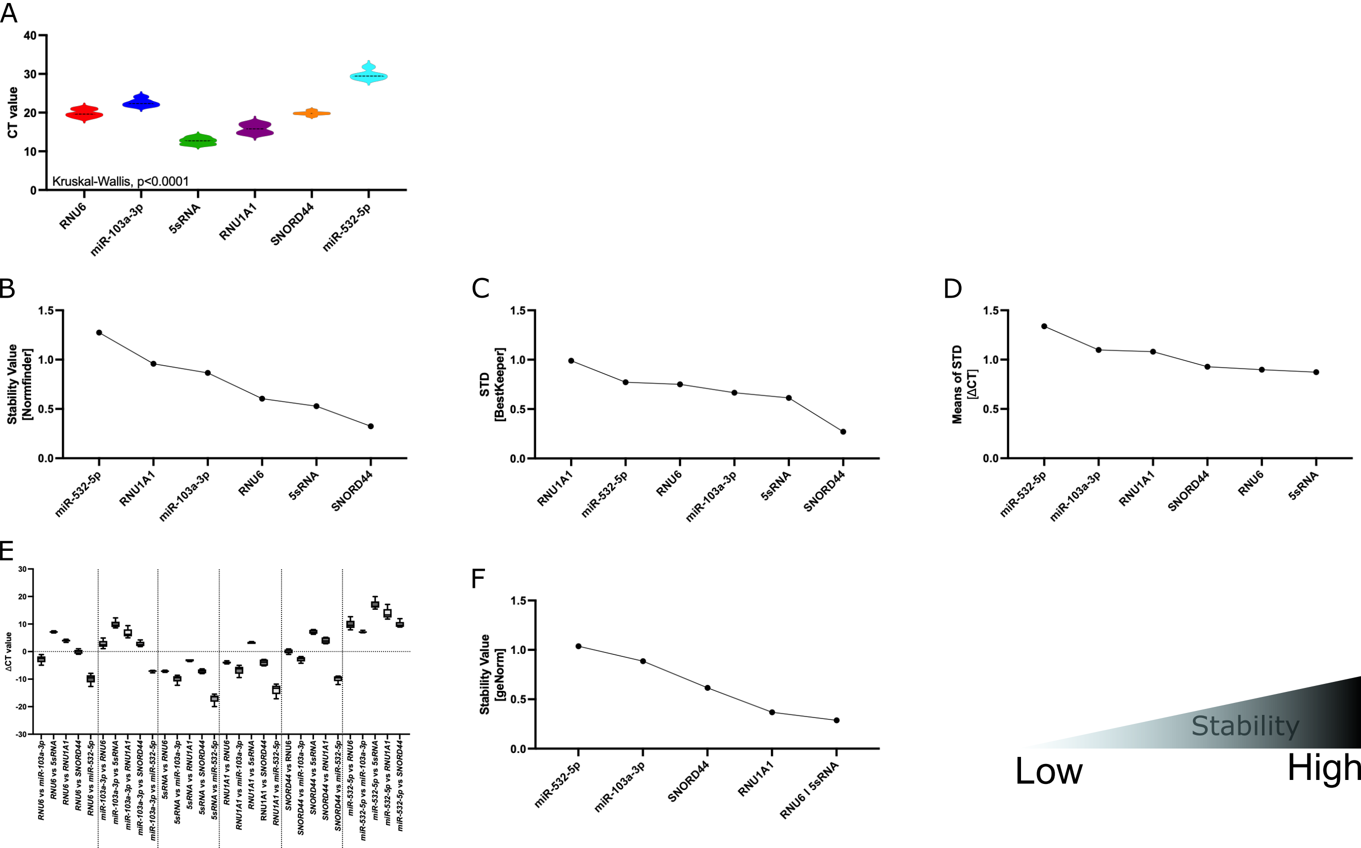

Supplementary Figure 4 Expression and stability of miRNA references in healthy controls of the validation cohort. (A) CT values of miRNA references within the validation cohort of healthy controls. The Normfinder (B), BestKeeper (C), ∆CT (D and E), or geNorm algorithm (F) were applied to assess gene stability. For A-D and F: decreasing values indicate increasing stability. E: ∆CT calculations based on CT[reference 1] – CT[reference 2]; mean CT values with min to max whiskers are shown.

Supplementary Figure 5

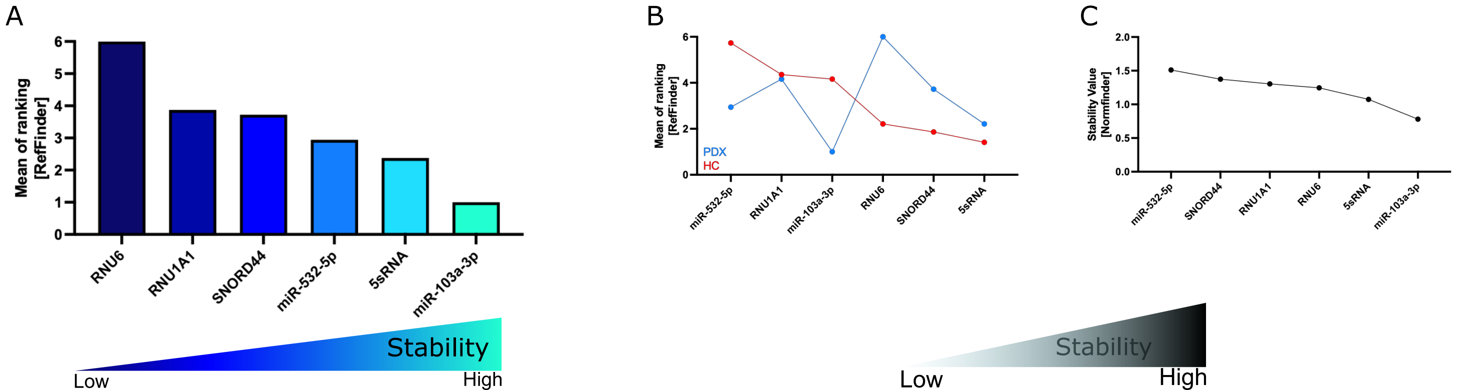

Supplementary Figure 5 Influence of sample type on miRNA reference stability in the validation cohort. (A) RefFinder values (mean of ranking; according to the Normfinder, BestKeeper, geNorm, and ∆CT algorithms) of the combined cohort of HCs and PDX ALL samples of the validation cohort (PDX ALL samples including spleen, BM, and CNS). (B) RefFinder values of PDX ALL specimens and HCs are depicted in Figure 10 and Figure 11 in a combined graph. (C) Normfinder results applying grouping (HCs and PDX ALL specimens) to the combined samples of the validation cohort. Decreasing values indicate increasing stability. BCP ALL: B-cell precursor acute lymphoblastic leukemia; PDX: patient-derived xenograft; HC: healthy control.

Supplementary Figure 6

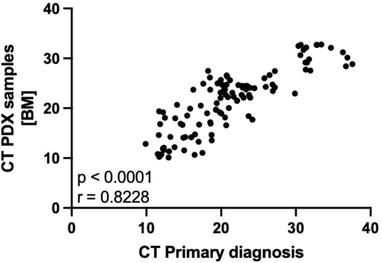

Supplementary Figure 6 Correlation of miRNA reference expression between PDX ALL samples and primary patient material. Primary patient material used to establish the mouse models was available for 23 of the 35 PDX ALL samples analyzed in this study. MiRNA reference expression of these samples was assessed and correlated to the expression levels of corresponding PDX ALL samples derived from BM. Spearman correlation. PDX: patient-derived xenograft; ALL: acute lymphoblastic leukemia; BM: bone marrow.
